# Zinc Finger Protein 82 regulates p53 protein stability through histone deacetylase and enhances neo-adjuvant chemotherapy in esophageal cancer

**DOI:** 10.1038/s41419-025-07979-1

**Published:** 2025-10-06

**Authors:** Weiyan Peng, Hongpeng Wang, Xuejuan Sun, Zhong Xu, Lingxiang Zhang, Lin Ye

**Affiliations:** 1 Department of Endocrine and Breast Surgery, Chongqing Key Laboratory of Molecular Oncology and Epigenetics, The First Affiliated Hospital of Chongqing Medical University, Chongqing, China; 2 Thyroid Oncology Department of Chongqing University Cancer Hospital, Chongqing, China; 3 Department of Cardiothoracic Surgery, The First Affiliated Hospital of Chongqing Medical University, Chongqing, China

**Keywords:** Cancer, Predictive markers

## Abstract

Tumor suppressor genes silenced by CpG methylation uncover the molecular mechanism of tumorigenesis and potential tumor biomarkers. Our previous research found that the promoter of zinc-finger protein 82 (ZFP82) was highly methylated in multiple cancers, including esophageal cancer, which induces the occurrence and development of tumors. Here, we describe the frequent detection of methylation of the ZFP82 promoter CpG Island in patients who did not respond to neoadjuvant chemotherapy, indicating that ZFP82 may related to esophageal cancer chemo-resistance. We further verified that in esophageal cancer cells expressing wild-type p53, ZFP82 bound to the HDAC3 promoter and mediated its interaction with p53, leading to HDAC3 cleavage and reduction of p53 ubiquitin-dependent proteasomal degradation, thus enhancing wild-type p53 stability. In cells expressing mutant p53, ZFP82 interacted with HDAC3 to regulate the down-regulation of HSP 70, leading to degradation of mutant p53. Through both mechanisms, the restoration of ZFP82 enhanced the chemosensitivity in esophageal cancer cells expressing wild-type p53 or mutant p53, significantly inhibiting in vivo tumorigenicity of these cells. Analyses of the expression of ZFP82 and clinical data indicated that ZFP82 expression correlated with improved prognosis. Our results define a mechanism for p53 stabilization via ZFP82-dependent HDAC3 decay under genotoxic stress conditions and validate a candidate bio-marker of early prediction of patients who will respond to esophageal cancer neoadjuvant chemotherapy.

## Introduction

The high mortality and low overall survival rates of esophageal cancer highlight the need to improve the treatment response [1, 2]. Neo-adjuvant chemotherapy before surgery is one of the classical therapeutic strategies for late-stage esophageal cancer [3, 4]. However, some patients do not respond to neo-adjuvant chemotherapy, so the overall survival (OS) rate of esophageal cancer remains as low as 15–20% [5, 6]. Understanding the mechanisms of chemotherapy resistance is important to improve the treatment of esophageal cancer.

The chemo-resistance of esophageal cancer involves multiple mechanisms. Aberrant DNA methylation of tumor suppressor genes was recently discovered to be related to the tumorigenesis and chemo-resistance of esophageal cancer [7–10]. Our group has studied the gene methylation profile of esophageal cancer, and determined that Zinc-Finger Protein 82 (ZFP82) was highly methylated in multiple cancers, including esophageal cancer [11]. We also demonstrated that ZFP82 induced apoptosis of esophageal cancer cells [12]. These observations have implicated ZFP82 as a potential therapeutic target for esophageal cancer. Recently, we collected tissue samples from esophageal cancer patients who experienced pathological complete response (pCR) and non-responders (NRs), after neoadjuvant chemotherapy and used the Infinium Methylation EPIC Bead Chip system (Illumina, San Diego, CA, USA) to analyze the DNA methylation profile of the two groups. The findings suggested that ZFP82 was a highly methylated gene in the tissue from NR.

Previous studies have shown that the histone deacetylase complex is the downstream target gene of the zinc finger protein family [13]. Protein structure analysis of histone deacetylase 3 (HDAC3) revealed that domains in the N-and C-terminal could interact with the Krueppel C2H2 structure of zinc finger family proteins [14, 15]. Zinc finger protein family member 521 (ZNF521) and promyelocytic leukemia zinc finger (PLZF) protein bind to HDAC3 through the Krueppel C2H2 zinc finger domain [16*–*18]. As a member of the zinc finger protein family, ZFP82 has a similar Krueppel C2H2 zinc finger domain that can bind to HDAC3 [11].

HDAC3 is a class I histone deacetylase. Its most important function is to catalyze the deacetylation of histone and non-histone proteins; accordingly, the gene encoding HDAC3 is one of the most frequently up-regulated genes in human cancer cells [19***–***21]. Chemotherapy resistance of tumors is partly due to the abnormal high-expression of HDAC3 [20***–***22]. Remarkably, HDAC3 can deacetylate the most important tumor suppressor, p53, in the human body. This leads to the degradation of wild-type p53 (WTp53) and loss of its inhibitory effect on tumor growth [23*–*25].

In this study, we found that the tumor-specific downregulation of ZFP82 is mediated by promoter methylation in patients who were non-responsive to neoadjuvant chemotherapy. Indeed, we demonstrated that ZFP82 exerts tumor suppressor function by inducing wild-type p53 acetylation through directly mediating the dissociation of HDAC3 from p53 and inducing the degradation of mutant p53. Furthermore, we reveal that ZFP82 is required for p53 stabilization to enhance esophageal cancer chemosensitivity.

## Methods

### Cell lines

Esophageal cancer cell lines KYSE960 (WTp53), TE6 (mutp53^R248Q^) (cell lines were purchased from RIKEN (BRC cell bank, Ibaraki, Japan), ATCC (American Type Culture Collection, Manassas, VA, USA) and were recently authenticated by short tandem repeat (STR) profiling and tested negative for mycoplasma contamination. All cells were cultured in RPMI 1640 medium supplemented with 10% (v/v) fetal bovine serum (FBS, Biological Industries, Israel) and antibiotics (100 units/mL penicillin and 100 μg/mL streptomycin). Cells were incubated at 37 °C in a humidified atmosphere containing 5% CO_2_.

### Tissue samples and evaluation of response to neo-adjuvant chemo-therapy

Fresh tumor tissues from non-responders to neo-adjuvant chemotherapy group (NR) and Pathological Complete Response group (pCR) were obtained from patients who underwent esophagectomy or biopsy under gastroscopy at the Department of Cardiothoracic Surgery in the First Affiliated Hospital of Chongqing Medical University (Patient clinical features were listed in Table 1). Investigators were blinded to group allocation during outcome assessment to minimize bias.

**Table 1:** List of the sequences used for in the study.

primer	Sense (5’ to 3’)	Anti-sense (5’ to 3’)
Msp		
ZFP82-m	TTTTTTTTAGGTTTTGTCGCGTC	CTACTAAAAAAACCGAACGCG
ZFP82-u	TTTTTTTTTAGGTTTTGTTGTGTT	CCAAACACACTCACAAAATACA
qPCR		
ZFP82	GAGCCTTGGAAAGTTGTGAG	GGCATTTTCACACTACTGAAG
HDAC3	CCTGGCATTGACCCATAGCC	CTCTTGGTGAAGCCTTGCATA
B-actin	TCCTGTGGCATCCACGAAACT	GAAGCATTTGCGGTGGACGAT
Chip		
ZFP82-1	CCTGGTGGCTCCCATCATTTA	CCCCATCTTTTCTGGTCCCT
ZFP82-2	CATGCTGGACCTCAGCATTTC	TGCTCAGGAAGCCTAGTGAT
ZFP82-3	AGCCCTTCTTCTTGAGCACG	GAGGACGGCATTCCTACCC
ZFP82-4	CACGAGATGGGACGGCATT	GGGAGGGGAGGAATCAAGGG
Bax	TAATCCCAGCGCTTTGGAAG	TGCAGAGACCTGGATCTAGCAA

Patients’ response to neo-adjuvant chemotherapy evaluation was based on the Response evaluation criteria in solid tumors (RECIST) Version 1.1. In general, computed tomography (CT) images were analyzed according to the following standards: all clinical and radiological evidence of the tumor showed a decrease of 30% or more in the sum of the longest diameters of all target measurable lesions, which were classified as the pCR group; An increase of more than 20% of the sum of the longest diameters of all target measurable lesions, the appearance of new lesions, or stable disease were classified as non-responders (NR). This research was approved by the Institutional Ethics Committees of the First Affiliated Hospital of Chongqing Medical University and followed the principles of the Declaration of Helsinki. Informed consent was obtained from each patient who participated in this study.

### Analyses using online databases

The UALCAN database (ualcan.path.uab.edu/) was used to analyze the correlation between ZFP82 expression and patient survival, the relationships between ZFP82 expression and ESCC patient clinical signatures and ZFP82 promoter methylation status in ESCC. The MethPrimer (https://methprimer.com/) was used to predict CpG islands. The STRING database (http://string-db.org/) was used to explored the possible related genes of ZFP82. The JASPAR database was used to predict the possible binding sites. The GEPIA database (http://gepia.cancer-pku.cn/) was used to analyze the correlation of genes. The threshold search value used for this study was a p-value < 0.05.

### Infinium MethylationEPIC BeadChip analysis

Infinium Methylation EPIC arrays (850k) were used to obtain genome-wide DNA methylation profiles of tissue specimens. Bisulfite treatment, whole-genome DNA amplification, hybridization and single-base extension, fluorescence staining and scanning of the chips were performed following the manufacturer’s instructions (Illumina).

### Establish chemo-resistance cells

5-FU resistance esophageal cancer cell lines KYSE960R (WTp53), TE6R (mutp53^R248Q^) were generated by exposing the cells to increasing concentrations of 5-FU (increased in a stepwise manner). In brief, cells were plated in six-well plates at a density of 1 × 10^5^ cells. Control cells were treated with vehicle alone (DMSO), model cells were treated with 5-FU (5, 10, 20, 40 μg /mL) and the cells were maintained in 5-FU (10 μg /mL). 3-D cell morphology, MTS assay and DNA damage markers were detected to identify the drug-resistance cell lines.

### RNA extraction

Cell lines and tissue samples treated with DNase I were used for RNA extraction by TRIzol® Reagent (Invitrogen, USA). The RNA concentration was measured by spectrophotometry and the storage temperature was -80 °C. RNA was reversely transcribed by Promega Reverse Transcription System (Promega, Madison, WI, USA). Semi-quantitative PCR was carried out using Go-Taq DNA polymerase (Promega, Madison, WI, USA) and reaction conditions were as previously reported [12]. Real-time PCR was performed using ABI SYBR green on an ABI 7500 Realtime PCR detection system (Applied Biosystems, Foster City, CA, USA) and conditions were as reported [12]. β-actin was used as a loading control. The primers used are listed in Table 1.

### BGS

Bisulfite modification of DNA and BGS were carried out as described [26]. For MSP, methylation-specific primers (annealing temperature 60 °C, 40 cycles) are listed in Table 1. MSP primers were confirmed previously for not amplifying any unbisulfite-treated DNA and, thus, specific to methylated DNA. For BGS, bisulfite-treated DNA was amplified using BGS primers (annealing temperature 60 °C, 40 cycles), and cloned into pCR4-TOPO vector (Invitrogen, USA). At least five colonies were randomly chosen for sequencing.

### Chromatin immunoprecipitation (ChIP) assay

2 × 10^8^ cells in 100-mm dishes were treated with PBS containing 1% formaldehyde for 10 min, washed with cold PBS and then incubated with 100 mM Tris (pH 9.4) and 10 mM DTT at 30 °C for 15 min. The cells were then rinsed twice in PBS and resuspended in 600 μL of Sol A. After a brief centrifugation, the cell pellets were resuspended in Sol B containing protease inhibitors followed by nuclear proteins extraction. After centrifugation at 13,000 rpm for 30 min, the nuclear pellets were resuspended in immunoprecipitation buffer and sonicated to into fragments of 0.5–1 kb average length. The ChIP assays were then preformed with the indicated antibodies. Primers were used for ChIP assays are listed in Table 1. All reactions were normalized relative to input activities and were presented as mean ± SD. Three independent experiments were repeated. The results are shown as a percentage of input.

### Plasmid and generation of stable cell lines

pcDNA3.1(+)-Flag-ZFP82 was generated as previously described [27], and its sequence was verified. To establish cell pools stably expressing ZFP82, full-length ZFP82 expression plasmid was transfected into KYSE960 cells, TE6 (mutp53R248Q) or KYSE960/R cells, TE6/R (mutp53R248Q) using the Lipofectamine 2000 system (Invitrogen, Carlsbad, CA, USA). The cells were maintained in 350 μg/ml of G418 for 14 days to establish stable cell lines and the ectopic expression of ZFP82 was confirmed by qPCR and western blot.

### Cell proliferation assay

Cell proliferation was assessed by MTS assay. Stable ZFP82-expressing cells and empty vector cells were seeded in 96-well plates (3000 cells/well) with 100 µL of medium. Cells were incubated for 24, 48 or 72 h. Subsequently, 20 µL MTS diluted in 100 µL/well serum free media was applied to each well. After a further 2 h incubated at 37 °C, absorbance was measured at 490 nm through a microplate reader (Multiskan MK3, Thermo Fisher Scientific). For each group, data from five different wells were pooled. All the experiments were performed in triplicate.

### Flow Cytometry analysis of apoptosis

For apoptosis, cells were transiently transfected with 4 μg pcDNA3.1-ZFP82 or empty vector as previously described, and then analyzed using Annexin V-FITC/PI staining. Data were analyzed using CellQuest™ Pro (BD Biosciences, San Jose, CA, USA).

### Three-dimensional culture

Cell 3-D culture was carried out as described by Bissell’s protocol [28]. Briefly, 120 µl/well Matrigel (BD Matrigel, TM, USA) was coated on pre-cooled 24-well plates, and incubated at 37 °C for at least 30 min. 4 × 10^4^ per well cells were diluted in culture medium with 10% Matrigel. Cell mixture was seeded onto the solidified gel surface. After incubation for 24 h, the cells in the entire well were fixed with 4% PFA and images were taken.

### Immunoprecipitation and Immunofluorescence staining

For Immunoprecipitation, Cell lines stably expressing vector or pcDNA3.1-ZFP82 were washed with ice-cold PBS three times and treated with lysis buffer. 0.5-1 mg of total protein in a total cell lysate volume of 500 μl was used for an IP reaction. 5 μg of primary antibody and 50 μl of magnetic beads were added to the tube and incubate at 4 °C overnight to form an antigen-antibody complex. Then, a western blot to was performed to detect the IP results. Protein samples were separated by SDS-PAGE, transferred to polyvinylidene difluoride membranes, and immune-stained according to the manufacturer’s instructions. Bands were detected using an ECL detection system. For co-immunoprecipitation, cells were fixed with 4% PFA and permeabilized with 0.1% Triton X-100. Cells were blocked for 1 h with 1% bovine serum albumin and incubated with primary antibodies 4 °C overnight. The primary antibodies used in this study were used as follows: DNA-PKcs (#38168, CST, USA), PI3K (#4249, CST, USA), Phospho-PI3K (#17366, CST, USA), HDAC3-N (#85057, CST, USA), HDAC3-C (sc-81600, Santa Cruz, USA), GAPDH (#2118, CST, USA), Caspase 3 (#9662, CST, USA), cleaved Caspase 3 (#9661, CST, USA), Caspase 5 (sc-393346, Santa Cruz, USA), BAX (#2772, CST, USA), p53 (#2524, CST, USA), HSP70 (#4872, CST, USA), PUMA (#98672, CST, USA), ZFP82 (PA5-36011, USA), GAPDH (sc-47724, Santa Cruz, USA), Actin (sc-8432, Santa Cruz, USA), acetyl-p53 (#2525 s, CST, USA), PUMA (sc-374223, Santa Cruz, USA), FAS (sc-8009, Santa Cruz, USA), FAS-L (sc-19681, Santa Cruz, USA). Full and uncropped western blots were uploaded as Supplemental Material.

### Bisulfite treatment and methylation-specific PCR (MSP)

Genomic DNA was extracted using the QIAamp DNA Mini Kit (Qiagen, Hilden, Germany) according to the manufacturer’s instructions. Bisulfite modification of DNA was conducted using an EZ DNA Methylation-Gold Kit (ZYMO Research, Irvine, CA, USA). MSP was performed as previously described [23]. MSP amplification of the bisulfite-treated DNA was performed at an annealing temperature of 60 °C for 40 cycles using the methylation-specific primer set for the ZFP82 promoter shown in Table 1. AmpliTaq Gold DNA Polymerase (Applied Biosystems, USA) was used to perform MSP and PCR products were identified on 2% agarose gels.

### HDAC activity assay

5 mg of histone H4 tail peptides were incubated with 0.25 mCi of [3H] acetyl coenzyme A (Amersham) in 20 mL of reaction buffer containing 50 mM Tris and 10 mM sodium butyrate at 30 °C for 2 h. Immunoprecipitated HDAC3 samples were incubated with 3H-acetate-labeled H4 histone in 150 mL of reaction buffer overnight at room temperature. The reactions were quenched with 1 M HCl and 0.16 M acetic acid. Released 3H-acetic acid was extracted with 600 mL ethyl acetate by vertexing and centrifugation (1 min at 9300 × *g*). The ethyl acetate supernatants (300 mL) were quantified by scintillation counting. The results are shown as mean ± SD, which were calculated from three independent experiments.

### TUNEL assay

The terminal deoxynucleotidyl transferase dUTP nick end labeling TUNEL Assay Kit-BrdU-Red (ab66110, Abcam, UK) was used to determine the number of apoptotic and necrotic cells according to the manufacturer’s instructions. TUNEL assay images were captured in a blinded manner and counted using flow cytometry.

### RNA sequencing

RNA sequencing (RNA-Seq) was conducted by BGI Tech Solutions Co., Ltd (China). The integrity of the RNA was evaluated using an RNA Nano 6000 Assay Kit on the Agilent Bioanalyzer 2100 system (Agilent Technologies, Santa Clara, CA, USA). For the construction of complementary DNA (cDNA) libraries, a total of 3 μg of RNA per sample was used as input material, and their quality was subsequently assessed using the Agilent Bioanalyzer 2100 system. The libraries were sequenced on the BGISEQ-500 RNA-seq platform, generating 50-base pair single-end reads. Genes exhibiting differential expression were identified using DESeq, with statistical significance defined as an adjusted p-value < 0.05.

### Animals

The mice used in this study were fed in the Animal Center of Chongqing Medical University. They were bred and treated in accordance with the national and regional ethical guidelines. All experiments were performed by certified personnel and approved by the Animal Use and Care Committee of the Animal Center, Chongqing Medical University. All mice were housed under a 12-h light/dark cycle and had free access to water and chow. A priori power analysis was performed using G*Power to determine the required sample size (n = 5), with parameters set at 80% power, a significance level of 0.05, and an anticipated effect size of 1.2 standard deviations. No animals or samples were excluded from the analysis; all subjects met the inclusion criteria and completed the experimental procedures as planned.

### Subcutaneous tumorigenesis outsourcing in nude mice

BALB/c-Nude (5–6 weeks of age, 18–20 g) mice were housed in specific pathogen-free facilities at 18–22 °C and 50–60% humidity. To investigate the effects of HDAC3 and ZFP82 activation on tumor growth, mice were randomly assigned to experimental groups using a manual random number drawing method. Subcutaneous injection of luciferase-expressing tumor cells was then performed in each mouse. After 28 days, some mice were treated intraperitoneally with 5-FU (25 mg/kg) every 3 days. To assess the self-renewal abilities of KYSE960/R cells in the tumorigenicity model, tumor lengths (L) and widths (W) were measured weekly using a digital caliper, and tumor volumes (V) were calculated using the formula V = L × W^2^/2. The maximal tumor size was permitted by the ethics committee, and the maximal tumor size was not exceeded.

For bioluminescent imaging assay, 15 minutes prior to imaging, mice were injected intraperitoneally (i.p.) with 150 mg/kg luciferin. Following general anesthesia, images were taken and analyzed with Spectrum Living Imaging System.

### Statistical analysis

All data represent three independent experiments and are presented as mean ± SD. SPSS 16.0 software was used for statistical analyses. Levene’s test confirmed equal variances, and Student’s t-test was used to determine significance. For all tests, *p* < 0.05 was considered statistically significant.

## Results

### ZFP82 is highly methylated and downregulated in chemo-resistant esophageal cancer patient tissues, and its methylation status correlates with patients’ chemosensitivity and outcome

**Fig. 1:**
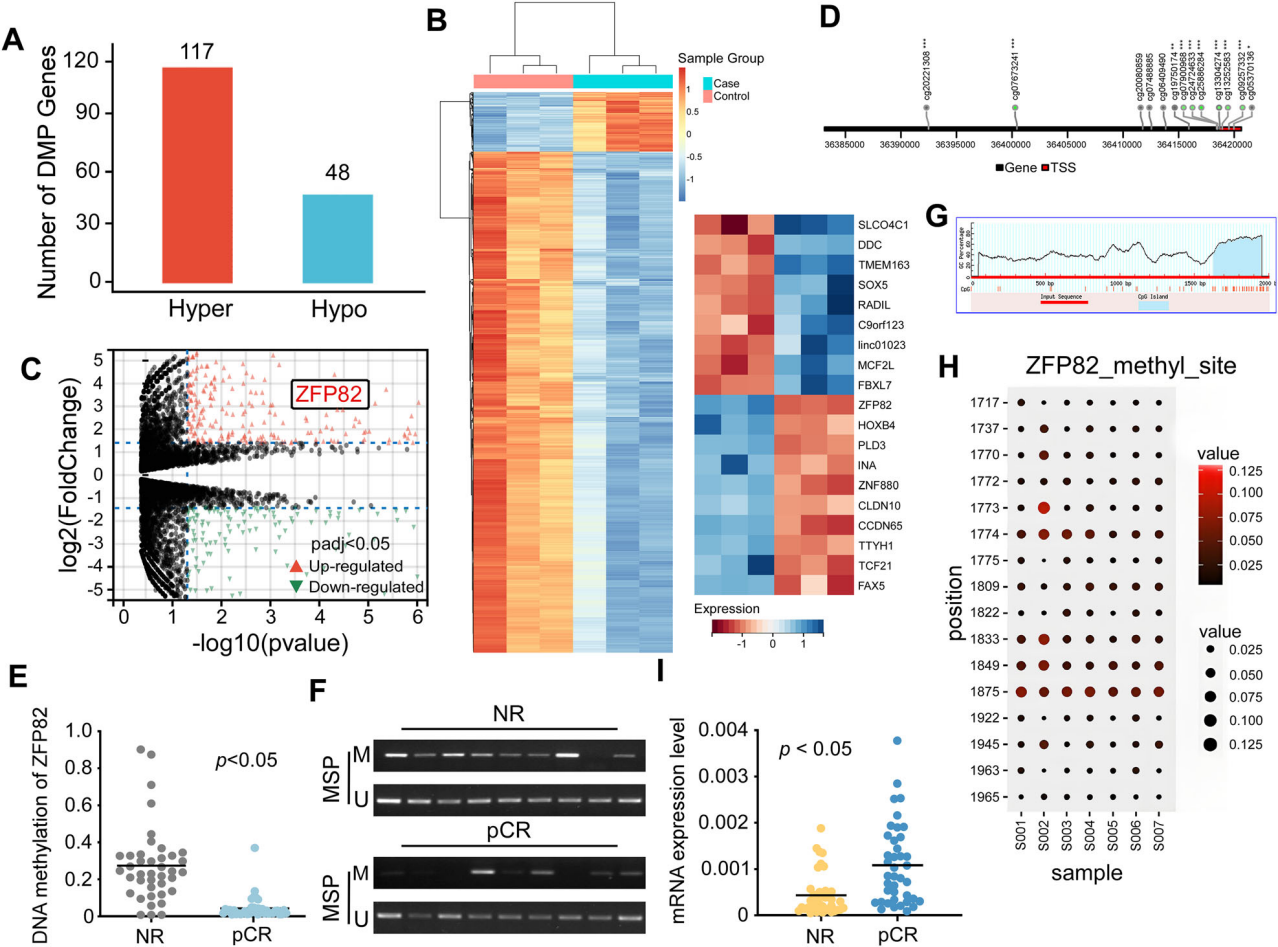
ZFP82 is highly methylated and downregulated in chemo-resistant esophageal cancer patient tissues. **A** Number of hypo-methylated and hyper-methylated gene in chemotherapy non-responder and pathologic complete responder esophageal tumor tissue samples through Infinium Human Methylation 850 Bead Chip. **B** Heatmap demonstrated ZFP82 is a highly methylated gene in non-responder tissues through Infinium Human Methylation 850 Bead Chip. **C** Volcano plot of hypo-methylated and hyper-methylated gene through Infinium Human Methylation 850 Bead Chip. **D** Differentially methylated probes within the ZFP82 promoter region were detected, with the green dot indicating a significantly hypermethylated region. **E** ZFP82 promoter methylation status by qMSP in NR and pCR esophageal tumor tissues (*p* < 0.05). **F** Representative image of MSP image in NR and pCR esophageal tumor tissues, M, methylated; U, unmethylated. **G** A CpG island in the ZFP82 promoter region was predicted using MethPrimer. **H** Methylated ZFP82 promoter alleles in three esophageal cancer cell lines (KYSE960/R, TE6/R and KYSE150/R) and four NPC tissue samples to confirm the result of MSP by Bisulfite genomic sequencing (BGS). **I** Differential mRNA expression of ZFP82 in chemotherapy non-responder and responder esophageal tumor tissue samples by semi-quantitative RT-PCR. NR chemotherapy non-responder, pCR pathologic complete responder.

We collected 40 tissue samples from patients and divided them into the NR and pCR groups, in accordance with RECIST Version 1.1. Patients’ details are shown in Table 2. The Infinium Human Methylation 850 Bead Chip was used to analyze the tissues from three NR and three pCR patients (Fig. 1A, B). In our previous research, we identified ZFP82 as a tumor suppressor gene in esophageal cancer [12]. Furthermore, when performing methylation analysis on samples from NR and pCR patients, we found that ZFP82 is also highly methylated in the NR group (Fig. 1C, D). To further verify the methylation status of *ZFP82*, methylation specific PCR (MSP) was performed in both groups. The results indicated that *ZFP82* was highly methylated in the NR group (33/40) compared to the pCR group (12/40) (Fig. 1E, F). To identify the specific methylation sites in the ZFP82 promoter region, we used MethPrimer to predict a CpG island located approximately 1700-2000 bp upstream of the ZFP82 promoter (Fig. 1G). We then analyzed the detailed methylation profile of this region in three esophageal cancer cell lines and four esophageal cancer tissue samples using bisulfite genomic sequencing (BGS). The results showed that this CpG island was hypermethylated at nearly every CpG site, with the significant highly methylated sites shown in Fig. 1H. In addition, we studied the expression of *ZFP82* mRNA by qPCR in both groups. *ZFP82* was down-regulated in most NR samples (34/40), while down-regulation of *ZFP82* was significantly less frequent (6/40) in the pCR group (p < 0.05; Fig. 1I). The collective observations indicated that ZFP82 was a highly methylated and down-regulated gene in tissues of NRs, suggesting that its methylation may be associated with the lack of response of the patients to neo-adjuvant chemotherapy. These findings suggested that the methylation status of *ZFP82* served as a potential prognostic factor for predicting the response of esophageal cancer patients to neo-adjuvant chemotherapy.

**Table 2:** Clinicopathological features of ZFP82 methylation in ESCC.

Clinicopathological features	Number(n = 40)
Gender	
Male	32
Female	8
Age	
<60	2
60-69	27
≥70	11
Phase	
Ⅰ	3
Ⅱ	14
Ⅲ	16
IV	7
Outcome	
alive	14
dead	26
Pathological classification	
squamous cell carcinoma	33
adenocarcinoma	7
Response to Neoadjuvant chemotherapy	
NR (non-responders)	23
pCR (pathological complete response)	17

### ZFP82 enhanced chemosensitivity in both WTp53 and mutation p53 (mutp53) expressing cell lines

**Fig. 2:**
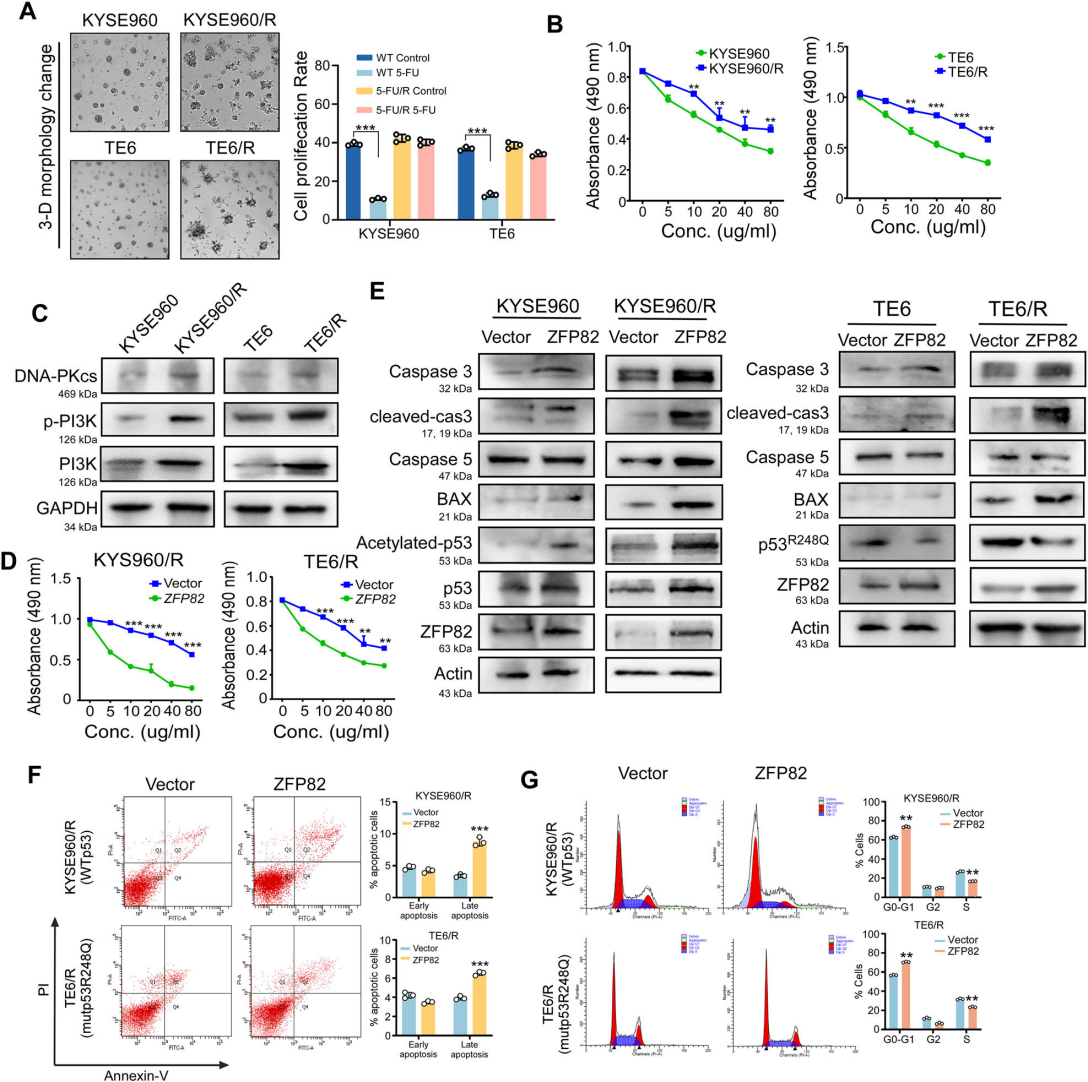
ZFP82 enhances esophageal carcinoma chemo-sensitivity in both WTp53 and mutp53 expressing cell lines. **A** 3-D cell morphology changes in 5-FU resistance esophageal cells line KYSE960R (WTp53), TE6R (mutp^53R248Q^) by cell 3-dementional culture (left). Cell viability was measured using the standard MTS assay (right). **B** MTS assay for cell proliferation on vector and resistance cell lines. The IC_50_ values for resistance cells were 38.97 vs 25.31 µg/mL in KYSE960/R cells and 46.35 vs 35.67 µg/mL in TE6/R cells. Asterisks indicate a significant level of proliferation compared with vector cells (**, *p* < 0.01; ***, *p* < 0.001). **C** Western blot indicated DNA-PKcs and phosphorylated PI3K were increased in resistance-cell lines KYSE960/R (WTp53), TE6/R (mutp53^R248Q^). **D** MTS assay for ectopic expression of ZFP82 and empty vector in drug-resistant cells, The IC_50_ values for Vector vs ZFP82 were 36.54 vs 10.73 µg/mL in KYSE960/R cells and 45.82 vs 18.89 µg/mL in TE6/R cells (**, *p* < 0.01; ***: *p* < 0.001). **E** Western blot showed in KYSE960 and KYSE960/R, ZFP82 induced cleaved caspase 3, caspase 3, 5 expressions, promoted the acetylation of p53 and induced the expression of BAX (left). In TE6 and TE6/R, ZFP82 has been demonstrated to upregulate the expression of cleaved caspase 3, while concurrently downregulating the expression of mutp53 (right). **F** Flow cytometry indicated that ZFP82 promotes apoptosis in KYSE960/R and TE6/R. A columnar statistical chart (***: *p* < 0.001) plotted based on the results of cytology. **G** Ectopic expression of ZFP82 blocks cell at G0-G1 phase in the presents of chemo-reagent 5-FU in both KYSE960/R and TE6/R (**, *p* < 0.01).

To explore the involvement of ZFP82 with chemoresistance persistence, we first induced resistance to 5-fluorouracil (5-FU) in esophageal cell lines KYSE960/R (WTp53), TE6/R (mut-p53^R248Q^). Tumor cell spheroids are considered a promising three-dimensional (3D) model in vitro widely used for drug resistance assessment. As shown in Fig. 2A, resistant cell lines formed spindle-like filopodia structures and microtubules. Cell viability was measured using the standard MTS assay (*p* < 0.05, Fig. 2A right). As shown in Fig. 2B, the half maximal inhibitory concentration (IC_50_) of 5-FU was 38.97 μg/mL in KYSE960/R cells vs. 25.31 μg/mL in control cells, and 46.35 μg/mL in TE6/R cells vs. 35.67 μg/mL in control cells. 5-FU is a synthetic fluorinated pyrimidine analog that causes cell death by incorporating the fluorinated product in DNA. DNA damage repair plays a crucial role in tumor chemoradiotherapy tolerance. The DNA-dependent protein kinase catalytic subunit (DNA-PKcs) serves as the catalytic subunits of DNA dependent proteases (DNA-PK) and belongs to the phosphatidylinositol-3-hydroxykinase (PI3K) family. It primarily participates in the repair of double-stranded DNA breaks and plays a role in cell apoptosis as well as tumor drug resistance. Western blot analysis indicated DNA-PKcs, p-PI3K and PI3K were increased in resistance-cell lines. These findings indicated that the cell lines we induced exhibited resistant to chemotherapy (Fig. 2C).

We overexpressed ZFP82 and empty vector in the resistant cell lines with WTp53 and mutp53. The results of the MTS assay validated the role of ZFP82 in chemotherapy sensitivity in drug-resistant cells, The IC_50_ values for Vector vs ZFP82 were 36.54 vs 10.73 µg/mL in KYSE960/R cells and 45.82 vs 18.89 µg/mL in TE6/R cells (Fig. 2D). This suggests that ZFP82 enhance chemosensitivity in both WTp53-expressing cells and mutp53-expressing esophageal cancer cells. Western blot analysis revealed that ZFP82 upregulated the expression of caspase 3, caspase 5, and BAX in both TE/R and KYSE960/R cells. Notably, ZFP82 enhanced p53 acetylation in KYSE960 and KYSE960/R cells while reducing mutp53 expression in TE6 and TE6/R cells. These effects are likely to play a role in promoting apoptosis in both parental and resistant cells (Fig. 2E). Flow cytometry indicated that ZFP82 promoted late apoptosis compared to the control group in both TE/R and KYSE960/R cells (Fig. 2F). Additionally, ectopic expression of ZFP82 blocked cells at the G0-G1 phase in the presence of 5-FU (10 μg/mL, *p* < 0.05, Fig. 2G). In KYSE960, ZFP82 overexpression may exert its effects by activating the p53 signaling pathway. In contrast, in mutant p53 cells, such as TE6, ZFP82 overexpression may function through alternative pathways to degrade Gain of function mutant p53, and may leads to G0-G1 cell phase arrest. Over all, these results indicated that ZFP82 enhanced the chemosensitivity in esophageal cancer cell lines expressing WTp53 or mutp53.

### ZFP82 enhances esophageal carcinoma chemosensitivity via interaction with HDAC3

**Fig. 3:**
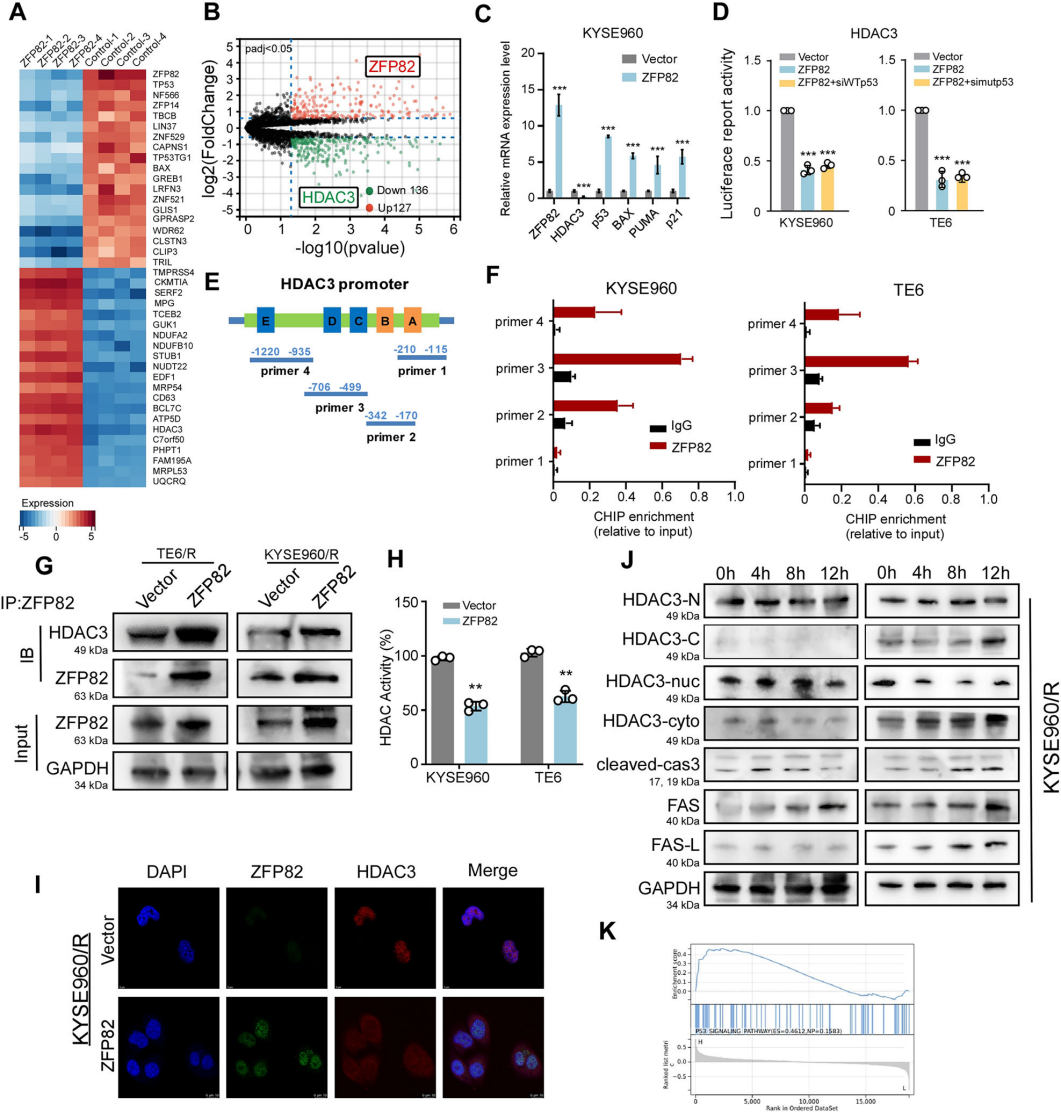
ZFP82 enhances esophageal carcinoma chemo-sensitivity through interacting with HDAC3*.* **A** RNA-seq shows in ZFP82 over-expressing cell lines compared to vector cells, significantly differentially expressed genes including molecules related to the p53 signaling pathway. **B** The volcano plot of differentially expressed genes includes HDAC3, which is significantly downregulated by ZFP82. **C** The regulatory effect of ZFP82 on HDAC3 and p53 signaling pathway genes was verified by qPCR. (**, *p* < 0.01). **D** Luciferase reporter assay results confirm that ZFP82 directly regulates the transcription of HDAC3. **E** Possible ZFP82-HDAC3 binding sites predicts by JASPAR database. **F** The binding of ZFP82 to the HDAC3 promoter was confirmed in both cell lines by ChIP-qPCR. **G** Identification of interaction between ZFP82 and HDAC3 by immunocoprecipitation assay. **H** ZFP82 inhibits HDAC3 activity by HDAC3 activity assay, (**, *p* < 0.01). **I** Ectopic expression of ZFP82 induced partial cytoplasmic translocation of HDAC3 in KYSE950/R cells as observed by immunofluorescence. **J** On ZFP82 over-expression KYSE950/R model, in the presence of chemo-reagent 5-FU (10 μg/mL), ZFP82 induced HDAC3 cleavage, caspases 3 cleavage, Fas, FasL expression in a time depended manner by Western blot (HDAC3-N antibody recognizes endogenous levels of total HDAC3 protein, HDAC3-C antibody raised against the C-terminus of HDAC3). **K** GSEA enrichment analysis shows that ZFP82 is related to the p53 signaling pathway.

To further explore the mechanisms by which ZFP82 enhanced the chemosensitivity of the esophageal cancer cells, RNA sequencing was performed in KYSE960 cells to screen for differentially expressed genes. The results revealed the significant down-regulation of the gene encoding HDAC3 (Fig. 3A, B). Recent studies have highlighted the connection between HDAC3 function and p53-mediated apoptosis. For instance, suppression of HDAC3 expression or inhibition of its activity increased p53 stability and acetylation in human cancer cells [29, 30]. RNA-seq analysis also revealed significant alterations in the expression of p53 pathway–related genes. Accordingly, we verified that ZFP82 regulates HDAC3 degradation and activates p53 downstream target genes in KYSE960 cells using qPCR (Fig. 3C). Furthermore, a luciferase assay confirmed that ZFP82 directly regulated HDAC3 transcription in both p53 wild-type and p53 mutation cell lines (Fig. 3D). Additionally, we knocked down WTp53 in KYSE960 cells and mutant p53 in TE6 cells, and observed that the expression of p53 did not influence the ability of ZFP82 to transcriptionally repress HDAC3 (Fig. 3D). To clarify the ZFP82-HDAC3 binding site, we first predicted the possible binding sites using the JASPAR database (Fig. 3E). Then, Chip-qPCR was applied to verify the binding of ZFP82 to the HDAC3 promoter in both cell lines (Fig. 3F). Previous studies have demonstrated that HDAC3 interacts with ZNF521 through its Krüppel-type C2H2 zinc finger domain, while ZFP82 possesses a structurally similar zinc finger domain [31]. Therefore, we further identified the interaction between ZFP82 and HDAC3 by co-immunoprecipitation (Co-IP) (Fig. 3G). The collective findings confirmed that ZFP82 and HDAC3 interacted with each other at both transcription and protein levels.

### ZFP82 induces HDAC3 cytoplasm translocation and inhibits both HDAC3 expression and activity

One of the main mechanisms of action of chemotherapy drugs is to destroy or damage the DNA of tumor cells, which is an important pathway to trigger cell apoptosis. Cleaved HDAC3 can be triggered and translocated from the nucleus to the cytoplasm, thus initiating the Fas/FasL apoptosis pathway [32***–***34]. Due to the cytoplasmic translocation of HDAC3, there is a decrease in HDAC3 binding to WTp53 in the nucleus, resulting in a decrease of HDAC3 induced p53 deacetylation and, in turn, increased stability of WTp53 [15]. We next examined the influence of ZFP82 on HDAC3 following their interaction. Given that the enzyme activity is a key function of HDAC3, we first evaluated the changes in HDAC3 activity after the overexpression of ZFP82 in cellular models. Expression of ZFP82 resulted in a decrease of HDAC3 histone deacetylase activity (Fig. 3H). Immunofluorescence analysis confirmed that expression of ZFP82 induced partial cytoplasmic translocation of HDAC3 in KYSE960/R. (Fig. 3I). Western blot analysis of ZFP82-overexpression KYSE960/R model revealed that in the presence of 10 µg/mL 5-FU, ZFP82 induced HDAC3 cleavage, increased cytoplasmic HDAC3, decreased nuclear HDAC3, and upregulated caspases as well as Fas and FasL expressions in a time-dependent manner (Fig. 3J). These findings indicate that ZFP82 interacts with HDAC3, inducing its cytoplasmic translocation and triggering Fas/FasL-mediated apoptosis.

### ZFP82 stabilizes p53 by inhibiting the interaction between HDAC3 and p53

**Fig. 4:**
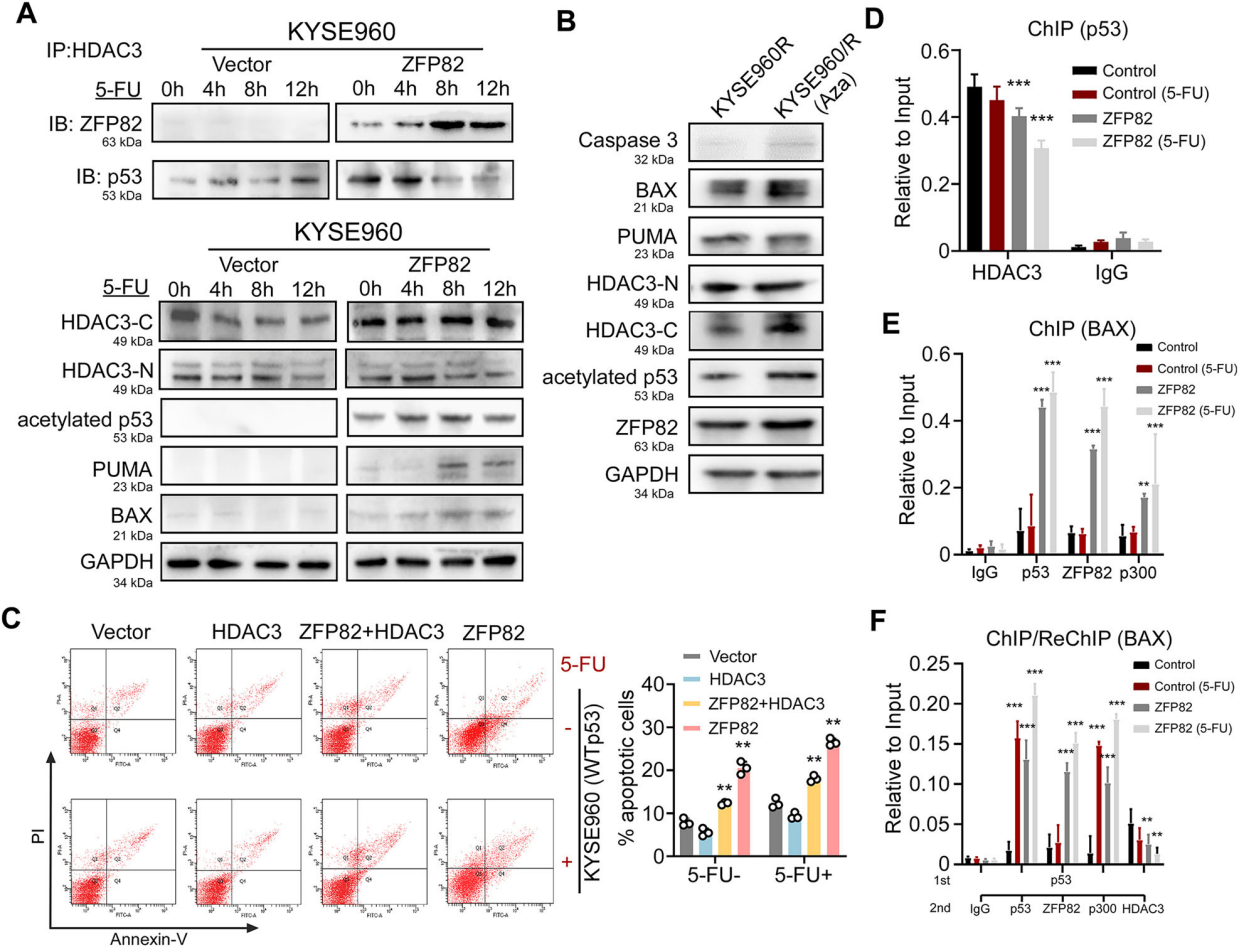
ZFP82 stabilizes p53 by inhibiting HDAC3–p53 interactions. **A** Upper panel: Immuno-coprecipitation suggests ZFP82 interacts with HDAC3 and disrupted HDAC3-p53 interaction in a time depended manner, in the presence of chemotherapy reagent 5-FU (10 μg/mL). Lower panel: In the presence of 5-FU (10 μg/mL), ZFP82 induces HDAC3 cleavage, and thus promotes p53 acetylation, down-stream target of p53 signaling PUMA and BAX is also activated. **B** After demethylation treatment of 5-Azacytidine (Aza), the restored expression of ZFP82 also induced p53 acetylation, HDAC3 cleavage, activates its downstream targets Puma and Bax, and promotes caspase-3 apoptosis pathway. **C** Flow Cytometry demonstrates in the presence of 5-FU (10 μg/mL), co-overexpression of HDAC3 + ZFP82 leads to partially restored apoptosis function, while overexpression of ZFP82 along significantly enhances the 5-FU induced apoptosis (HDAC3 vs HDAC3 + ZFP82 vs ZFP82: 9.32% vs 18% vs 26.52%, *p* < 0.05). right panel shows the statistic histogram of the apoptosis results (**, *p* < 0.01). **D** Chromatin Immunoprecipitation (ChIP) analyses against the p53 promoter. Compared to vector, whether at the presence of 5-FU or not, ectopic expression of ZFP82 decreased HDAC3-p53 complex on p53 promoter (**, *p* < 0.01). **E** ZFP82 increases p53-p300 complex to the promoter region of Bax. Chromatin Immunoprecipitation (ChIP) analyses against the p53 binding sites (p53-RE) of BAX promotor shows, compared to vector, whether at the presence of 5-Fu or not, the re-expressing of ZFP82 enhances 5-FU-induced recruitment of the p53-p300 complex to the p53-RE of Bax promotor (***, *p* < 0.001). **F** ChIP and re-ChIP assays showed that ZFP82 overexpression enhanced the efficiency of 5-FU–induced binding of acetylated p53 and p300 to the p53 response element of Bax, while reducing the binding of HDAC3 to p53. (***, *p* < 0.001).

Inhibition of HDAC3 induces deacetylation of p53 is an important mechanism for enhancing the stability of WTp53 and tumor cell chemosensitivity [19]. We obtained the GSEA software (version 3.0) from the GSEA website and divided the samples into high expression group (>=50%) and low expression group (<50%) based on the expression level of ZFP82. We also downloaded the c2.cp.kegg.v7.4.symbols.gmt subset from the Molecular Signature Database to evaluate the relevant pathways and molecular mechanisms. Based on gene expression profiles and phenotype grouping, we set the minimum gene set to 5 and the maximum gene set to 5000, with 1000 resamples, *p* < 0.05 and FDR < 0.25 were considered statistically significant. GSEA revealed that ZFP82 is related to the p53 signaling pathway (enrichment 0.4612, Fig. 3K). Thus, we explored whether ZFP82 inhibited HDAC3 induced WTp53 deacetylation and degradation, and promoted p53 acetylation and protein stability in the presence of 10 µg/mL 5-FU. First, the binding of HDAC3 and p53 was detected by Co-IP in cells ectopically expressing ZFP82 or vector after a 12-h exposure to 5-FU. ZFP82 disrupted the interaction between HDAC3 and p53, the p53-HDAC3 complex was decreased in a time-depended manner (Fig. 4A upper panel). Western blot revealed the inductions of p53 acetylation and HDAC3 cleavage, and the activations of the downstream target of p53 signaling PUMA and BAX (Fig. 4A lower panel).

Upon establishing that the silencing of ZFP82 in esophageal cancer cells was partially due to CpG island methylation, we next tested following the demethylation of 5-Azacytidine, the restored expression of ZFP82 induced HDAC3 cleavage and induced p53 acetylation, thereby activating p53 downstream targets (Fig. 4B). To further understand the function of ZFP82 on induction of HDAC3 cleavage, flow cytometry was performed. In the presence of 10 µg/ml 5-FU, overexpression of HDAC3 led to resistance to apoptosis, overexpressing of ZFP82 increased apoptosis rate, and co-overexpression of HDAC3 and ZFP82 partially restored cell apoptosis (Fig. 4C), suggesting that ZFP82 could disrupt apoptosis resistance induced by HDAC3 by inducing HDAC3 cleavage. Bradye et al. [35] reported that p53 mediates transcriptional activation upon binding to the promoter region of target genes, such as BAX. To further explore the specific mechanisms of ZFP82 in the inhibition of HDAC3 and activation of the p53 downstream apoptosis pathway, we next explored the possibility that ZFP82 formed a complex with HDAC3 and disrupted the interaction between p53-HDAC3 and thus enhanced p53 recruitment to the promoter region of its target genes. For this, we performed chromatin immunoprecipitation (ChIP) involving the p53 promoter and p53 binding elements on BAX. ChIP assay showed that ZFP82 could reduce the binding between HDAC3 and p53 in the presence of 5-FU (*p* < 0.01, Fig. 4D). Further ChIP and Re-Chromatin Immunoprecipitation (re-ChIP) analyses showed that ZFP82 promoted the binding of p53 and p300 to the p53 response element (p53-RE) of the BAX promoter. (*p* < 0.01, Fig. 4E, F). Collectively, these data demonstrated that ZFP82 mediated the removal of HDAC3 from binding to p53 upon genotoxic stress, and subsequently induced p53/p300 binding to the BAX promoter that in turn promoted the transcriptional activation of p53-target genes.

### ZFP82 inhibits HDAC3 and promotes mutp53 degradation

**Fig. 5:**
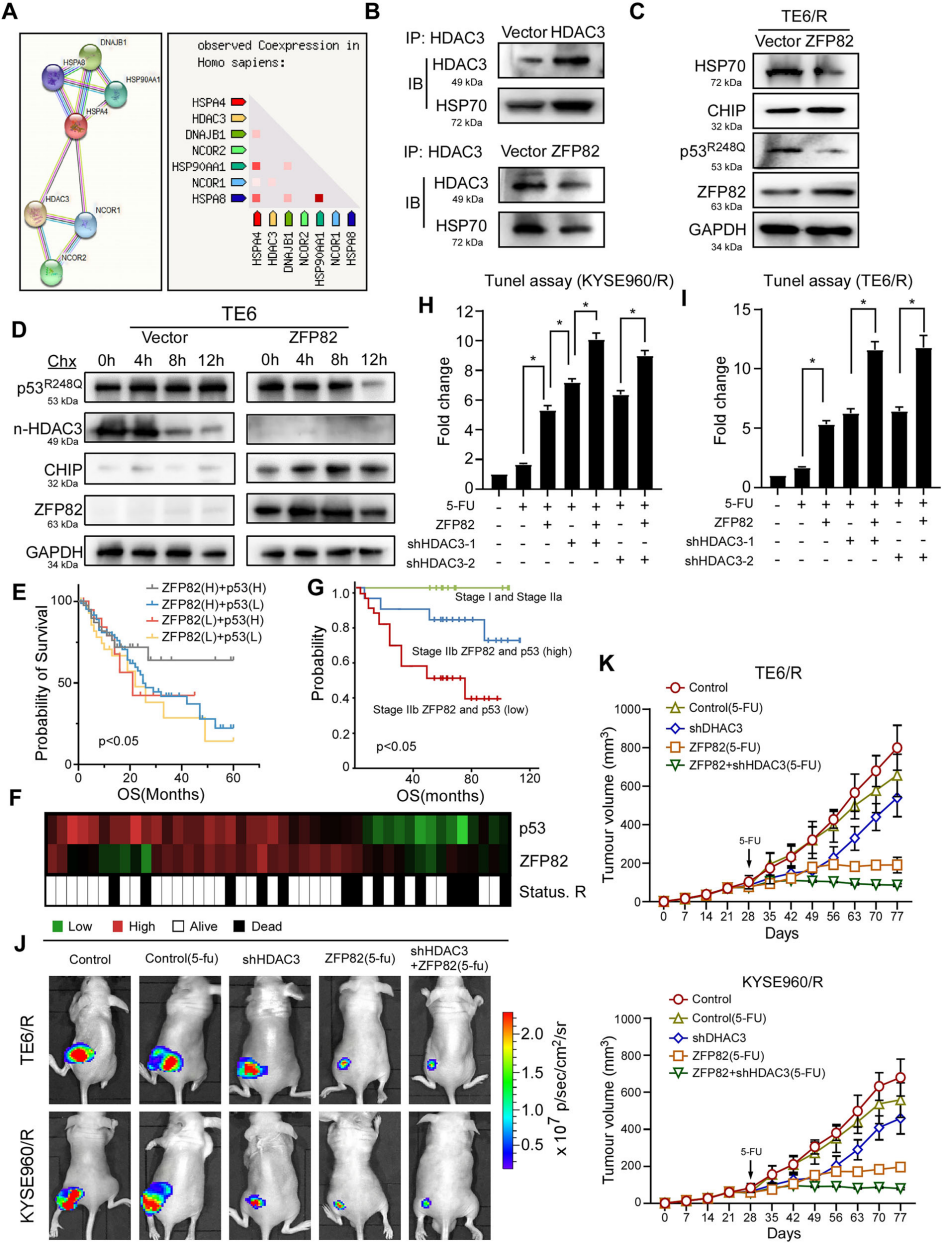
ZFP82 inhibits HDAC3 and promotes mutation p53 degradation. Reduction of ZFP82 and p53 induces esophageal tumorigenesis. **A** In String database (https://string-db.org), the nucleotide exchange factor of HSP70, HSPA4 is the downstream target of HDAC3. **B** Co-IP assay verifies the binding of HSP70 and HDAC3. Over-expression ZFP82 disrupted the HSP70-HDAC3 complex. **C** Western-blot verified in resistant TE6/R cell lines, ZFP82 induced CHIP expression and down regulated HSP70 and p53^R248Q^. **D** TE6/R cells were treated with CHX 10 μg/mL, and time course western blot suggests ZFP82 re-expression increased CHIP expression and induced p53^R248Q^ and HDAC3 degradation. **E** Survival analysis using TCGA database. The patients are sub-stratified into four groups, as the data shown, patients with high expression of both ZFP82 and p53 have the highest survival rate, *p* < 0.05. **F**, **G** The pathological correlation between ZFP82 expression and p53 in esophageal cancer was investigated. Patients were stratified into the following groups: stage Ⅰ, stage Ⅱa, stage Ⅱb (ZFP82 plus wtp53 (high)) and stage Ⅱb (ZFP82 plus wtp53 (low)). Tumor tissues from esophageal cancer patients were used to generate gene expression by qPCR. Data showed that patients with stage Ⅰ and stage Ⅱa were all survived within 5 years, stage Ⅱ patients with increased expressions of ZFP82 and p53 showed relatively good prognostic outcomes, with a 5-year survival rate exceeding 80%. The 5-year survival rate was < 50% for stage Ⅱ patients with low expressions of ZFP82 and p53 (log-rank test P¼ 0.00387). **H** Overexpress of ZFP82 and HDAC3 knockdown potentiates 5-FU-induced apoptosis. Stable ZFP82-expressing KYSE960/R cells were transfected with shHDAC3, and then treated with 5-FU. DNA damage of cells was determined by the TUNEL assay. * p < 0.01. **I** Overexpress of ZFP82 with HDAC3 knockdown potentiates 5-FU-induced cell apoptosis in TE6/R cells. Stable ZFP82-expressing TE6/R cells were transfected with shHDAC3, and then treated with 5-FU. DNA damage of cells was determined by the TUNEL assay. * p < 0.01. **J** In vivo tumorigenicity assay, KYSE960/R and TE/6 cells were subcutaneous injected into nude mice, representative bioluminescent images of subcutaneous tumor outgrowth are shown. In vivo tumor growth indicates that ectopic expression of ZFP82 or knockdown of HDAC3 significantly inhibits the tumor growth compared to vector, significantly increased chemo-sensitivity of KYSE960 cells to 5-FU. **K** The overexpression of ZFP82 substantially enhances the chemosensitivity of KYSE960/R and TE6/R cell lines. Cells were stably transfected and subsequently injected subcutaneously into the left flank of athymic nude mice. Following a four-week period post-inoculation, animals presenting with tumors of similar dimensions (ranging from 100 to 200 mm³) were selected for chemotherapy with 5-FU at a dosage of 25 mg/kg, administered with a 2-day interval over a period of 8 weeks. Tumor volume measurements were conducted over a 12-week duration. The error bars depicted in the corresponding figures represent the standard deviation of the mean.

Unlike WTp53, the stability of mutp53 is maintained by heat shock protein (HSP) families [36***–***38]. The HDAC inhibitor Suberoylanilide hydroxamic acid (SAHA) inhibits HDAC6 and inactivates HSP90 by inducing HSP90 over-acetylation through interacting with the C terminus of E3 ubiquitination ligase and carboxy-terminus of Hsp70-interacting protein (CHIP. CHIP specifically targets mutp53 instead of WTp53 [39]) [40]. Notably, like HSP90, HSP70 also serves as a critical regulator of CHIP-mediated ubiquitination and degradation of p63 isoforms, a homolog of p53 [41]. In brief, HSP70 inhibits CHIP expression and reduces the ubiquitination degradation of mutp53 [42], enhancing the stability of mutp53 [39] and reducing chemosensitivity of cancer cells. Scrutiny of the STRING database (https://string-db.org) revealed that the nucleotide exchange factor of HSP70, HSPA4 [43], was the downstream target of HDAC3 (Fig. 5A). Next, we used the Co-IP assay to verify the binding of HSP70 and HDAC3 (Fig. 5B, upper panel). Overexpression of ZFP82 disrupted the HSP70-HDAC3 complex (Fig. 5B lower panel). Western blot analysis of resistant TE6/R cells showed ZFP82 induced CHIP expression and downregulation of p53^R248Q^ (Fig. 5C). The functional effect of ZFP82 on mutp53 degradation was investigated by western blot analysis of TE6/R cells at different times following treatment with 10 µL of Cycloheximide (CHX). ZFP82 overexpression increased CHIP expression and induced p53^R248Q^ degradation, while also decreasing the nuclear HDAC3 in a time-dependent manner (Fig. 5D). These results indicated that in esophageal cells harboring mutp53, ZFP82 abolished the HSP70-HDAC3 complex and enhanced CHIP induced ubiquitination degradation of p53^R248Q^, thus increasing cell chemosensitivity.

### Reduction of ZFP82 and p53 induces esophageal tumorigenesis

We and others have described the downregulation of ZFP82, which had been reported in patients with gastric and breast cancer [11, 44]. To investigate the relationship between ZFP82 and WTp53, we analyzed the expression levels of ZFP82 and WTp53 in esophageal cancer patients using the GEPIA database (http://gepia.cancer-pku.cn). Next, we investigated the prognostic power of ZFP82 and WTp53. Neither ZFP82 nor WTp53 expression levels alone showed significant prognostic discrimination. However, the combined signature of ZFP82 and WTp53 showed strong prognostic powers in esophageal cancer (*p* < 0.05, Fig. 5E). We also investigated the pathological correlation based on the expression level of ZFP82 and WTp53 in esophageal cancer tumor samples. Tumor tissues from esophageal cancer patients were used to assess gene expression by qPCR. Gene expression profiles of ZFP82 and WTp53 in esophageal cancer patients revealed a strong positive correlation (Fig. 5F). We next stratified patients into the following groups: stage Ⅰ, stage Ⅱa, stage Ⅱb (ZFP82 and WTp53 (high)) and stage Ⅱb (ZFP82 and WTp53(low)). Patients with stage Ⅰ and Ⅱa all survived within 5 years. Stage Ⅱb patients with increased expressions of ZFP82 and WTp53 showed relatively good prognostic outcomes, with a 5-year survival rate exceeding 80%. The 5-year survival rate was < 50% for stage Ⅱb patients with low expressions of ZFP82 and WTp53 (Fig. 5G). Next, we confirmed the ZFP82-dependent apoptotic effects in response to chemotherapy stress using the KYSE960/R and TE6/R cell line by TUNEL assay. As shown in Fig. 5H, I, 5-FU exhibited minimal effects on the KYSE960/R cell line. Notably, HDAC3 knockdown combined with ZFP82 overexpression significantly enhanced the impact of 5-FU on the DNA damage response (Fig. 5H, I). Collectively, these data indicate that ZFP82 regulates anti-apoptotic action of HDAC3.

Next, tumorigenicity was assessed in vivo. KYSE960/R cells and TE6/R cells were subcutaneously injected into nude mice and tumor growth was observed. Knockdown of HDAC3 significantly increased the chemosensitivity of both KYSE960/R and TE6/R cells, indicating that HDAC3 inhibits p53-mediated genotoxic stress responses in esophageal cancer. ZFP82 overexpression significantly enhanced 5-FU chemosensitivity compared with the control group. Furthermore, co-overexpression of ZFP82 and knockdown of HDAC3 led to a greater increase in chemosensitivity than either treatment alone, further supporting a negative regulatory role of HDAC3 in p53-mediated genotoxic stress responses (Fig. 5K, J).

Collectively, these data demonstrated that ZFP82 inhibited in *vivo* tumorigenic growth of esophageal cancer cells, enhanced the chemosensitivity of tumor cells and correlated with better survival of esophageal cancer patients.

## Discussion

Esophageal cancer is a very aggressive tumor with increasing incidence. According to cancer statistics, with an estimated 511,000 new cases and 445,000 deaths in 2022, the highest rates are seen in Eastern Asia and Eastern Africa [45]. For locally advanced tumors, neoadjuvant chemotherapy has recently been utilized as an integral part of a multi-modal treatment strategy. This treatment provides a survival benefit for locally advanced patients when compared with surgery alone. However, approximately half of the patients show no obvious response to chemotherapy. Therefore, early prediction of NRs would be valuable and important to avoid inefficient therapy, toxic side effects, and additional costs. For esophageal adenocarcinoma, several studies have concentrated on the analysis of markers that are relevant to the response to chemotherapy [46]. Despite increasing knowledge regarding the molecular background of this cancer entity, no valid biomarkers have been identified to predict prognosis or neoadjuvant chemotherapy response in esophageal squamous cell carcinoma, which is the major esophageal cancer type in China.

We have previously used epigenetic analyses to identify many tumor suppressor genes in esophageal cancer [11]. Furthermore, an analysis using the Infinium Methylation EPIC BeadChip system revealed different methylation patterns between responders and NRs. The differences included ZFP82, a recently discovered tumor suppressor. Our research group previously used gene expression profiles and DNA methylation Co-IP to show that ZFP82 was downregulated by promoter CpG island methylation in a variety of malignant tumors [12]. It has been proposed that ZFP82 is a potential tumor suppressor for various malignant tumors, including esophageal and gastric tumors, by inducing tumor apoptosis [11, 44]. These previous results indicated that ZFP82 may be a potential therapeutic target for esophageal cancer. However, whether the altered expression of ZFP82 is related to esophageal cancer chemosensitivity is still unclear. We used RNA-seq to demonstrate that the gene encoding HDAC3 was down-regulated after ZFP82 overexpression. HDAC3 is a Class I histone deacetylase; its most important function is to promote the deacetylation of histone and non-histone proteins. The HDAC3 gene is one of the most frequently upregulated genes in human cancer cells, and its abnormally high expression is part of the reason for tumors’ resistance to chemotherapy [47].

Notably, in cells, HDAC3 can deacetylate p53, which is the most important tumor suppressor in the human body, leading to the degradation of WTp53 and loss of of tumor growth inhibition. Accumulating evidence indicates that the inhibition of HDAC3 enhances p53 acetylation and stability in human cancer and normal cell lines. Furthermore, a recent report showed that HDAC3 suppresses p53 activity in coordination with melanoma antigen gene-A, which confers resistance to chemotherapeutic agents [48].

WTp53 and mutp53 (35–84%) are widely present in esophageal cancer tissues. Among them, the majority of mutp53 are gain-of-function mutations [49]. In tumor cells that are resistant to chemotherapy, deacetylation catalyzed by HDAC3 reduces the stability of WTp53 and increases the stability of gain-of-function mutp53 [50]. Therefore, downregulating HDAC3 to promote the stability of WTp53 and degrade gain-of-function mutp53 through specific pathways is a potential molecular mechanism for reversing tumor chemotherapy resistance.

The present findings demonstrated that p53 function was reversibly regulated by the HDAC3-ZFP82 interaction during the genotoxic stress response. The stability of tumor suppressor WTp53 depends on post-translational modifications [51]. When chemotherapy drugs cause cellular DNA damage, the damage can trigger caspase-dependent C-terminal cleavage of HDAC3 and the nucleus-to-cytoplasm translocation of HDAC3. Both events initiate Fas/FasL-mediated apoptosis. Due to the cytoplasmic translocation of HDAC3, the binding between HDAC3 and p53 in the nucleus is reduced. The ensuing reduction in the deacetylation of p53 in the nucleus increases the stability of WTp53 [34]. Wang et al. reported that suppressing the deacetylation of WTp53 by inhibiting HDACs was an important mechanism for enhancing the stability of WTp53 and increasing chemosensitivity [52].

In this study, we demonstrated that *ZFP82* was a downregulated gene in esophageal cancer patients who did not respond to chemotherapy due to promoter CpG methylation at the ZFP82 loci. The MTS viability assay demonstrated enhanced chemosensitivity upon the ectopic restoration of ZFP82 in the resistant cell lines KYSE960/R (WTp53) and TE-6/R(p53R248Q). HDAC3 decreases cell apoptosis and induces cell S phase arrest in the absence and presence of 5-FU. In contrast, after HDAC3-ZFP82 co-transfection, ZFP82 restored cell apoptosis and induced cell cycle arrest in the G0/G1 phase by flow cytometry. These cell function analyses indicated that, to some extent, ZFP82 countered HDAC3-induced cancer cell growth.

We further analyzed the interaction between HDAC3 and ZFP82. RNA-seq revealed that HDAC3 was downregulated by ZFP82. The luciferase assay results confirmed that ZFP82 regulated HDAC3 transcription. The SimpleChIP Enzymatic Chromatin IP Kit was used to verify the binding site of ZFP82 and HDAC3 promoter. Co-IP findings further verified the binding of the two proteins. Immunofluorescence results indicated that ZFP82 induced the cytoplasmic translocation of HDAC3. The collective findings supported the view that ZFP82 bound to the HDAC3 promoter and induced the nucleus-to-cytoplasm transfer of HDAC3. Finally, western blot confirmed that the cytoplasmic translocation of HDAC3 enhanced p53 stability and induced the expression of downstream target of p53 signaling.

Intriguingly, ChIP-qPCR revealed that the interaction between ZFP82 and HDAC3 reduced the binding of HDAC3 to p53, and led to the formation of p53/p300 complex. Re-ChIP showed, in the p53-RE of BAX, that ZFP82 increased the recruitment of p53 to the p53-RE of BAX when forming the p53-p300 complex, and decreased the HDAC3 binding to the BAX promoter. This finding suggests that the interaction of ZFP82, HDAC3, and p53 is required for the binding of p53 to the target gene promoters, and this association promoted the transcription of p53 downstream pro-apoptotic genes.

Conversely, unlike WTp53, the stability of mutp53 is mainly determined by the heat shock protein 70 (HSP70) and HSP90 families [38]. HSP70 stabilizes mutp53 by binding to the C-terminus of the carboxy-terminus of Hsp70-Interacting Protein (CHIP) E3 ubiquitination ligase. The binding inhibits CHIP expression and reduces the CHIP catalyzed ubiquitination-mediated degradation of mutp53. CHIP directly targets mutp53, not WTp53 [39].

The HDAC inhibitor SAHA inhibits HDAC6, and inactivates HSP90 by excessive acetylation [53]. This leads to the ubiquitination-mediated degradation of mutp53 and enhances chemosensitivity [40]. Co-IP results demonstrated the binding of HDAC3 and HSP70 in TE-6/R cells. Overexpression of ZFP82 downregulated HSP70 and increased CHIP expression via the inhibition of HDAC3, which in turn decreased the stability of mutp53 and increased the chemosensitivity of esophageal cancer cells.

Finally, our clinical analyses confirmed a negative correlation between ZFP82 and HDAC3, and esophageal cancer patient survival rates. Survival rates of stage Ⅱ esophageal cancer patients with reduced levels of ZFP82 and p53 were markedly decreased compared with those of patients with a high level of ZFP82 and p53 expression. This result is consistent with that from the xenograft assay. This suggests that reduction of HDAC3 and increased ZFP82 levels may contribute to inhibiting the early progression of esophageal cancer.

The use of HDAC inhibitors to enhance the effect of chemotherapy has been extensively reported, and several inhibitors are currently in clinical trials. Recent studies in the development of selective HDAC inhibitors have implicated HDAC3-selective inhibitors as promising candidate anticancer drugs. The present study demonstrated that ZFP82 interaction with HDAC3 enhanced chemosensitivity in the treatment of esophageal cancer. Future efforts to develop clinical applications for HDAC inhibitors may focus on the use of HDAC subtype-specific inhibitors and tumor suppressors that target specific HDAC subtypes. A better understanding of the regulation of tumor suppressors on specific HDAC functions will pave the way for the development of HDAC inhibitors as a means of enhancing cancer chemotherapy.

## Conclusions

**Fig. 6:**
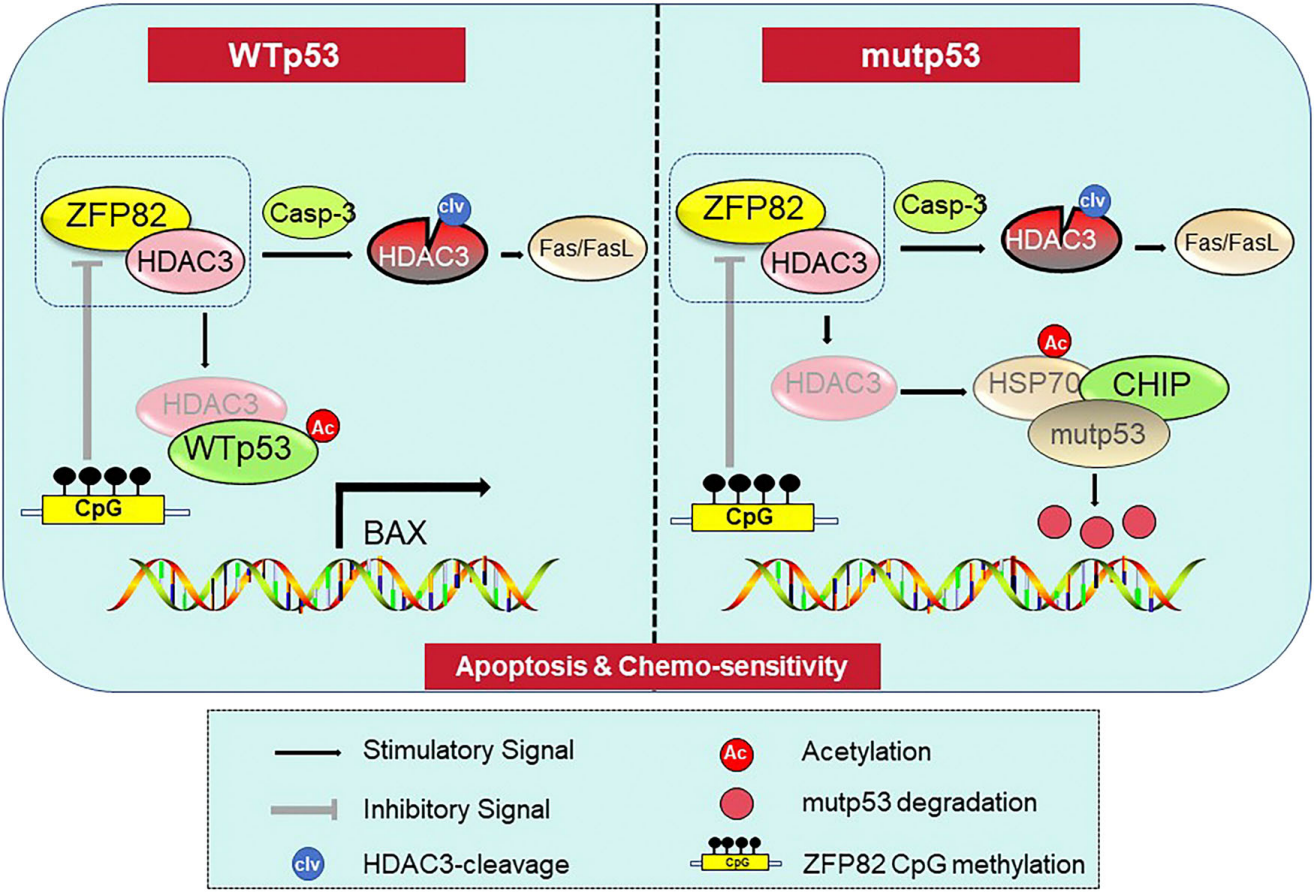
The schematic diagram of the mechanisms how ZFP82 regulates p53 protein stability through interacting with histone deacetylase 3 in cell expressing wild type p53 and mutant p53 in esophageal cancer.

ZFP82 was highly methylated in non-responders to esophageal cancer neoadjuvant chemotherapy compared to those who achieved pathological complete response by Infinium Methylation EPIC Bead Chip. The mechanisms for this may be related to HDAC3; RNA-seq demonstrated that HDAC3 may be a direct target of ZFP82. HDAC3 is a Class I histone deacetylase; its most important function is to promote the deacetylation of histone and non-histone proteins, including p53. ZFP82 induced the nucleus-to-cytoplasm transfer of HDAC3 and enhanced p53 stability and induced the downstream target of p53 signaling, and finally enhanced the chemosensitivity of esophageal cancer. This provides evidence for using ZFP82 as a biomarker for the early prediction of esophageal cancer patients who respond to chemotherapy (in Fig. 6. the schematic diagram of the mechanisms of ZFP82 shows that it regulates esophageal cancer chemosensitivity by interacting with HDAC3).

Importantly, our experiments were limited since we could not obtain a larger patient sample. Our in vitro study was also limited in its capacity to reproduce the complexity of the multiple interacting cell types in vivo. Furthermore, the in vivo study did not involve the use of gene knockout mice, due to budget and the time limitations. Nevertheless, a benefit of our approach is that the cells used were homogenous, facilitating the generation of controlled experiments, and reproducible and robust experimental data.

## Supplementary information

Full and uncropped western blots

Full and uncropped western blots

Full and uncropped western blots

Full and uncropped western blots

Full and uncropped western blots

Full and uncropped western blots

Full and uncropped western blots

Full and uncropped western blots

Full and uncropped western blots

Full and uncropped western blots

Full and uncropped western blots

Full and uncropped western blots

## Data Availability

The datasets generated and/or analyzed during the current study are available from the corresponding authors on reasonable request.
