# Supplementary figures and images for: Zinc Finger Protein 82 regulates p53 protein stability through histone deacetylase and enhances neo-adjuvant chemotherapy in esophageal cancer
Source: Cell Death Dis. 2025 Oct 6;16(1):694. doi: 10.1038/s41419-025-07979-1 (PMC12501244; doi:10.1038/s41419-025-07979-1)

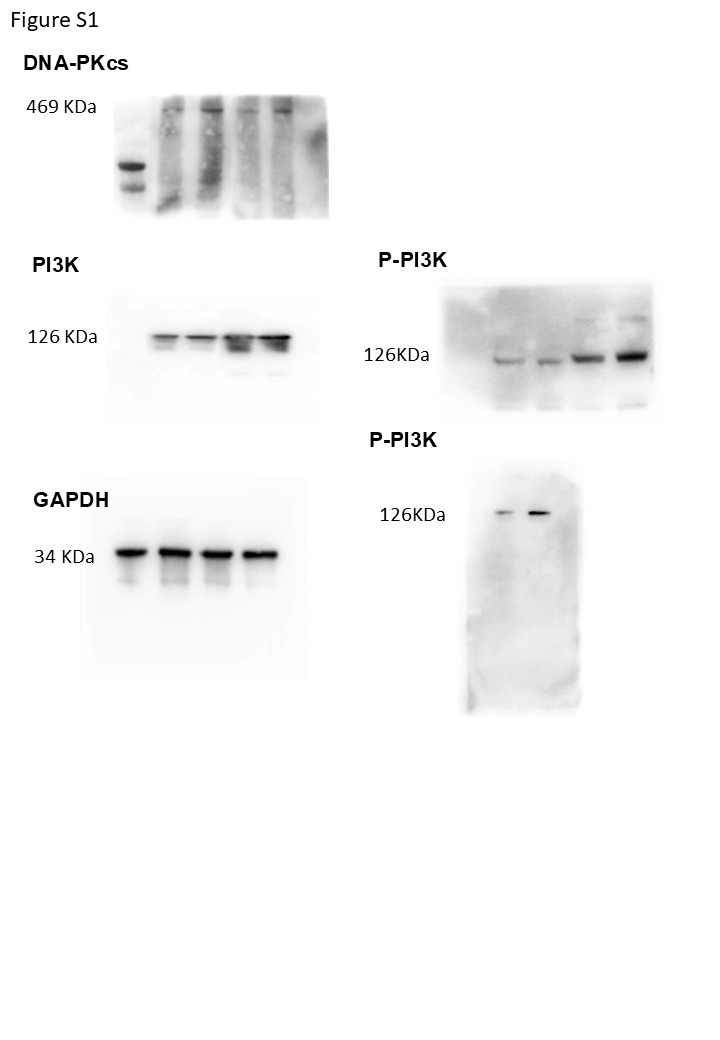

Supplement: Supplementary file 1 — Full and uncropped western blots [file 41419_2025_7979_MOESM1_ESM.jpg]

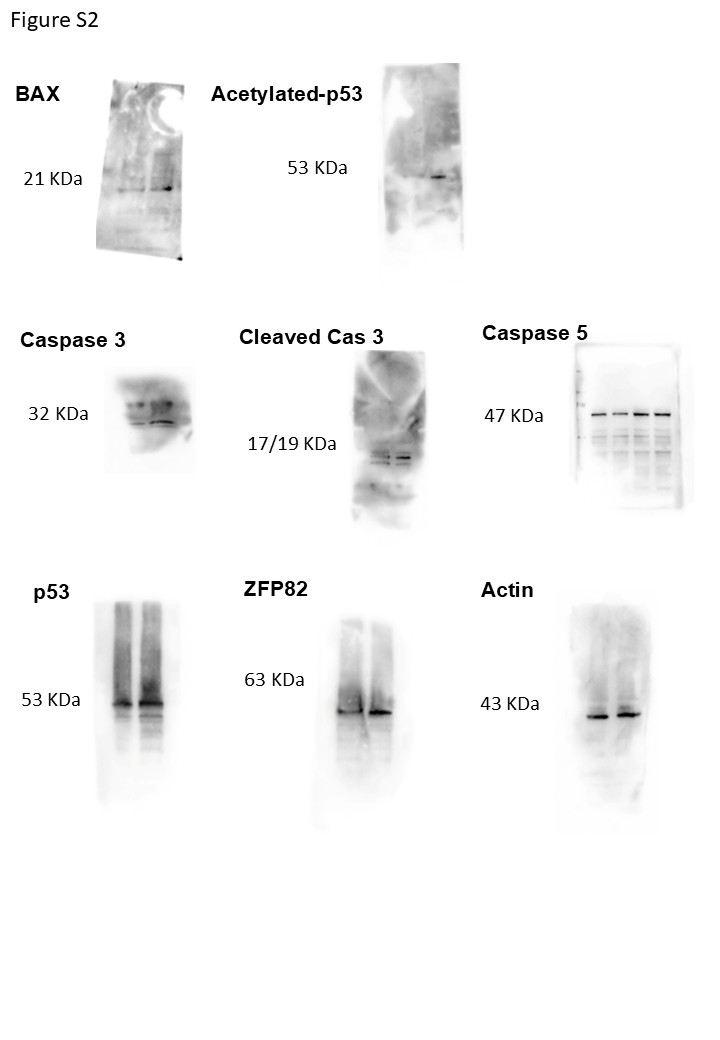

Supplement: Supplementary file 2 — Full and uncropped western blots [file 41419_2025_7979_MOESM2_ESM.jpg]

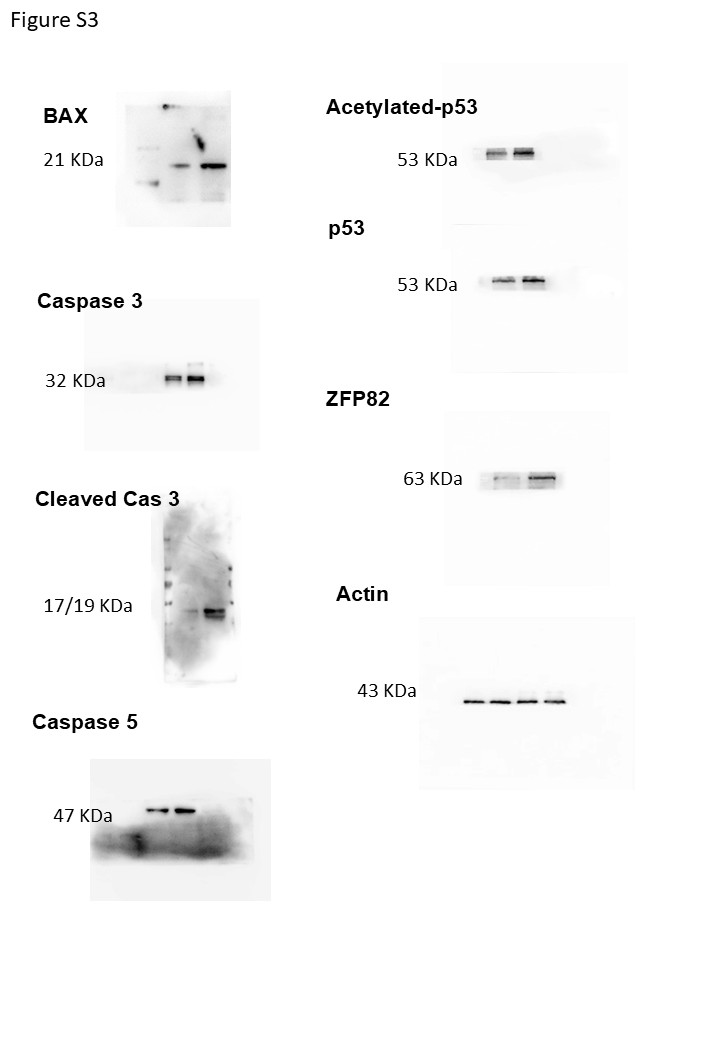

Supplement: Supplementary file 3 — Full and uncropped western blots [file 41419_2025_7979_MOESM3_ESM.jpg]

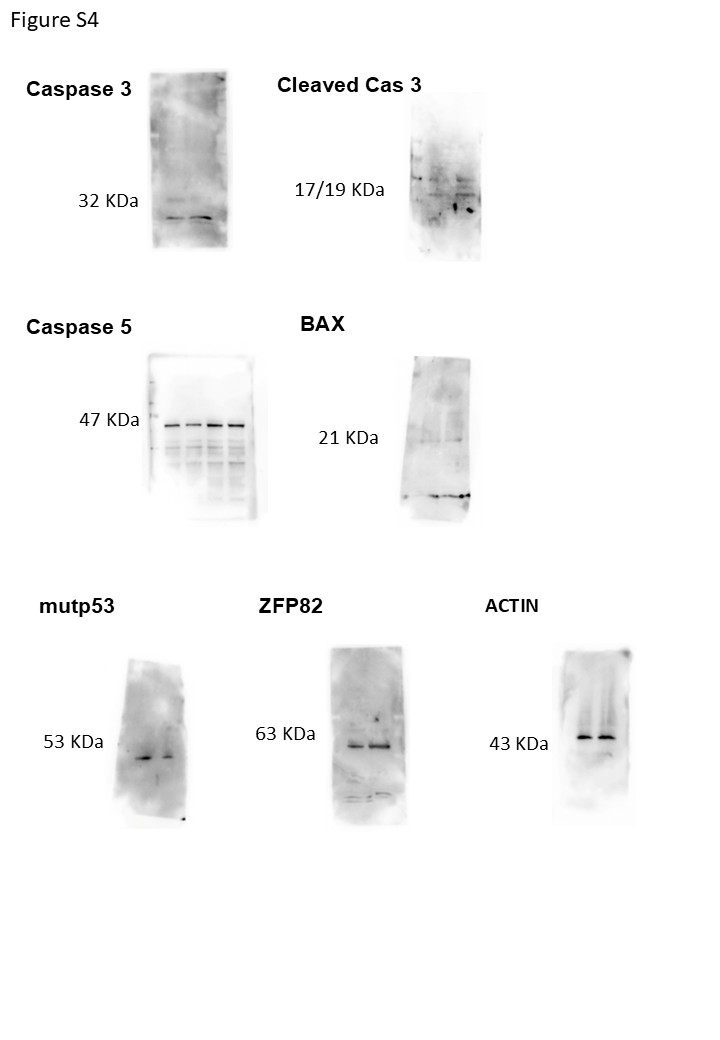

Supplement: Supplementary file 4 — Full and uncropped western blots [file 41419_2025_7979_MOESM4_ESM.jpg]

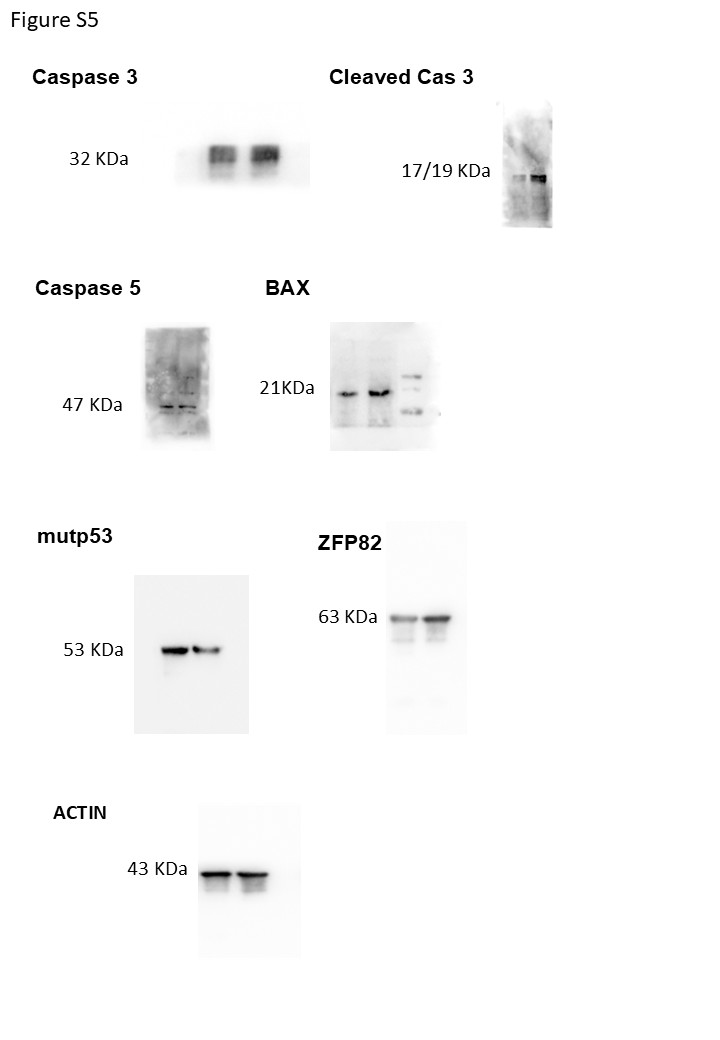

Supplement: Supplementary file 5 — Full and uncropped western blots [file 41419_2025_7979_MOESM5_ESM.jpg]

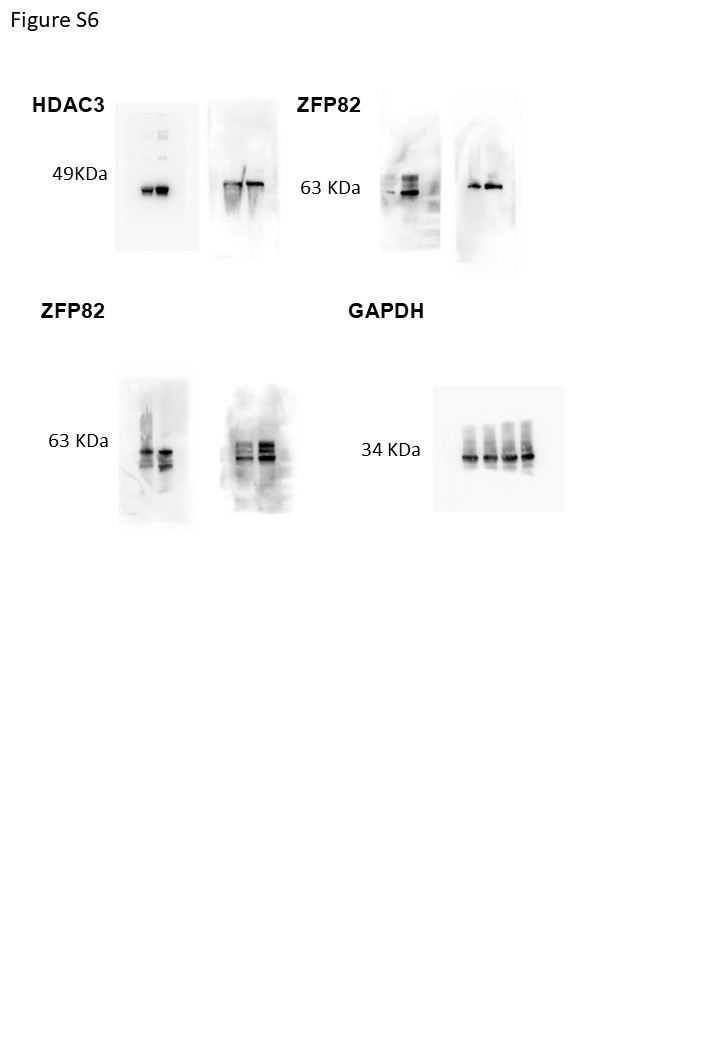

Supplement: Supplementary file 6 — Full and uncropped western blots [file 41419_2025_7979_MOESM6_ESM.jpg]

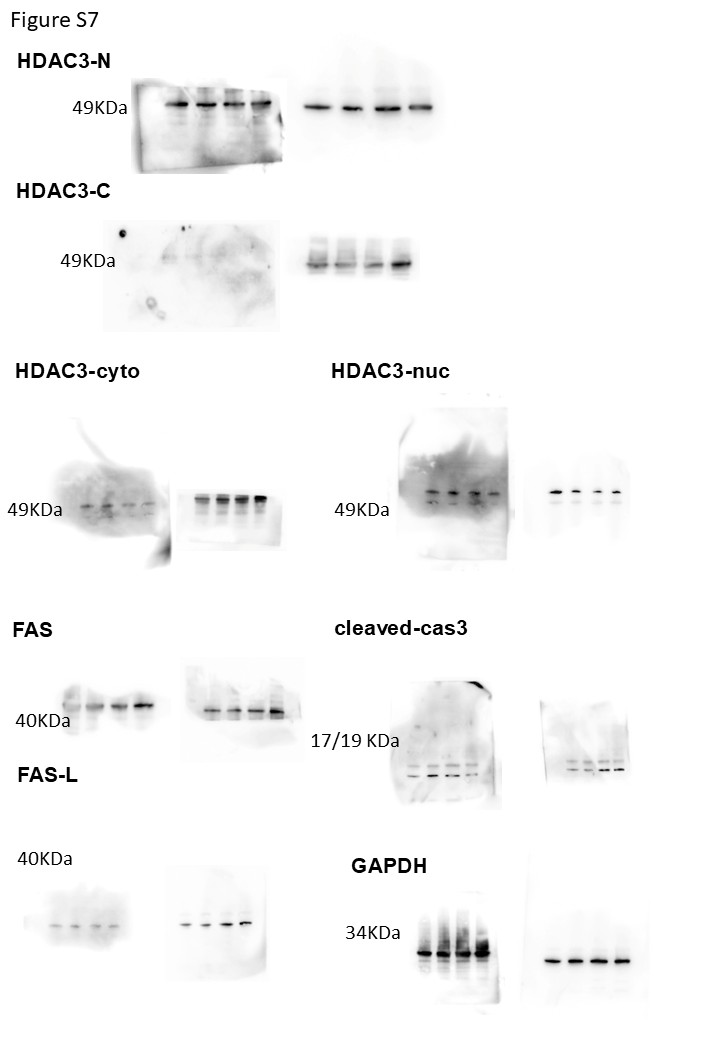

Supplement: Supplementary file 7 — Full and uncropped western blots [file 41419_2025_7979_MOESM7_ESM.jpg]

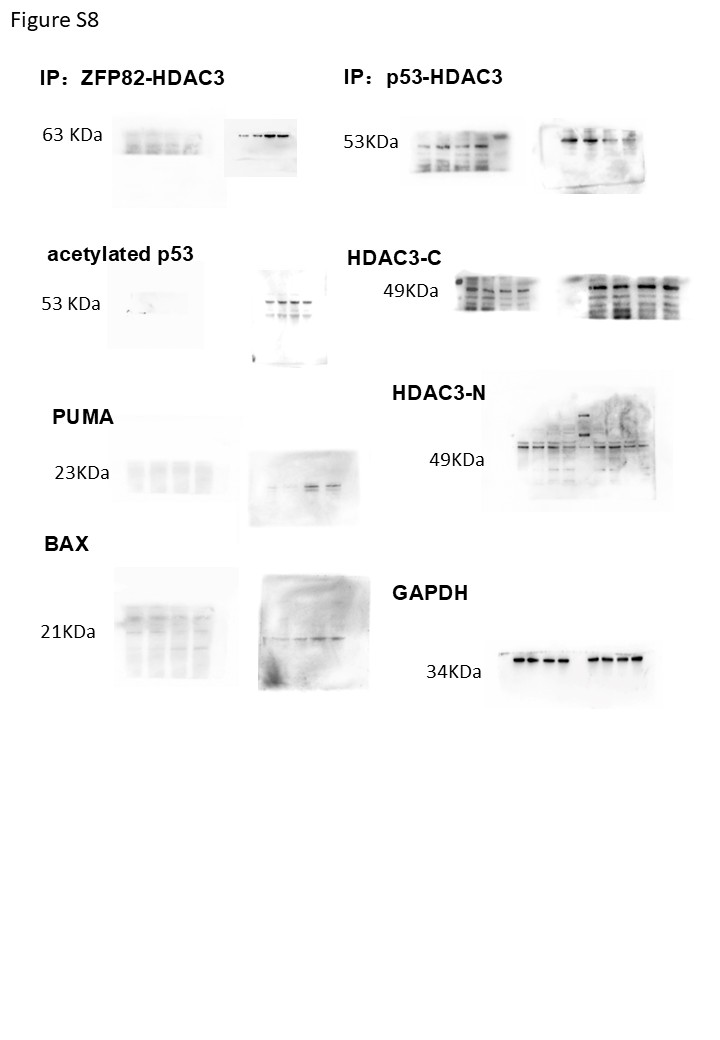

Supplement: Supplementary file 8 — Full and uncropped western blots [file 41419_2025_7979_MOESM8_ESM.jpg]

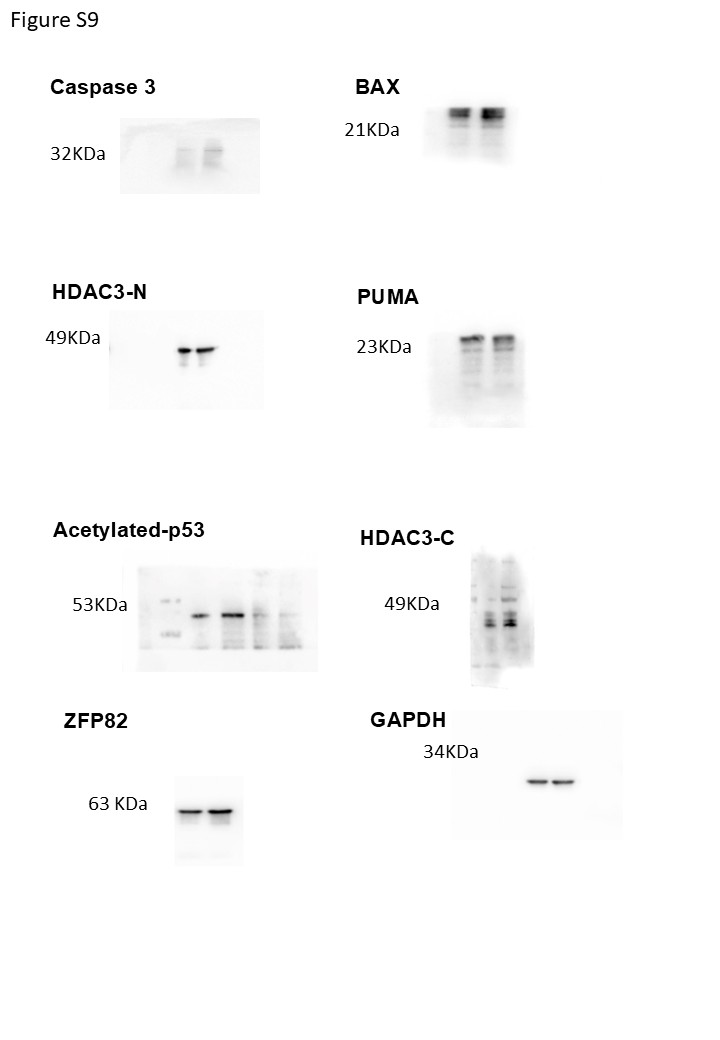

Supplement: Supplementary file 9 — Full and uncropped western blots [file 41419_2025_7979_MOESM9_ESM.jpg]

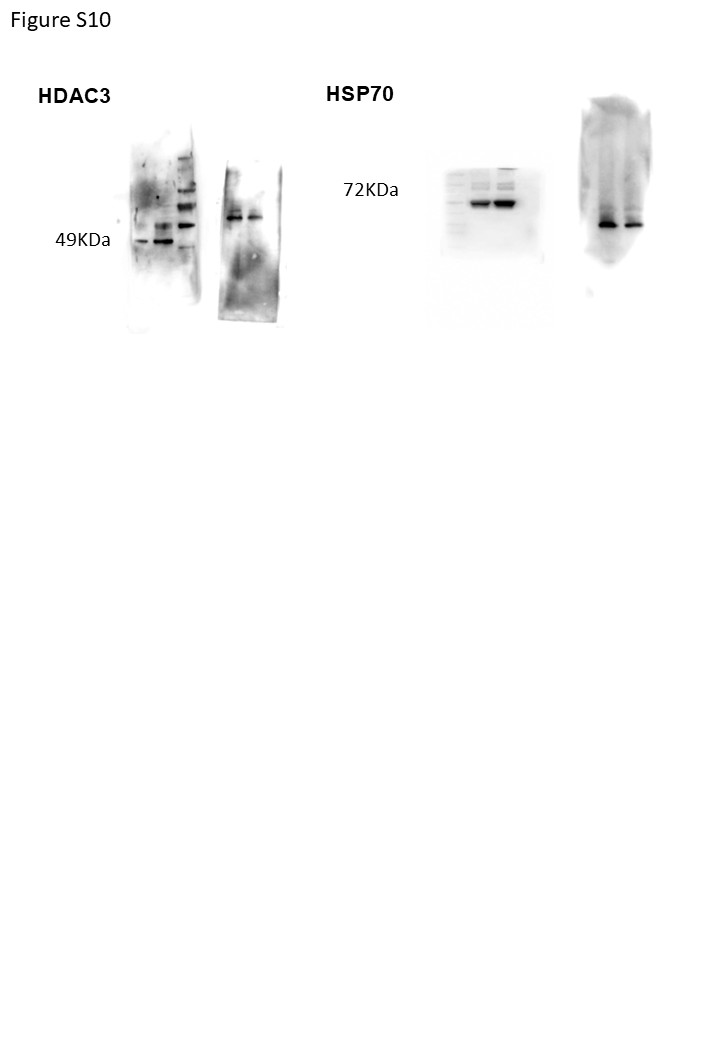

Supplement: Supplementary file 10 — Full and uncropped western blots [file 41419_2025_7979_MOESM10_ESM.jpg]

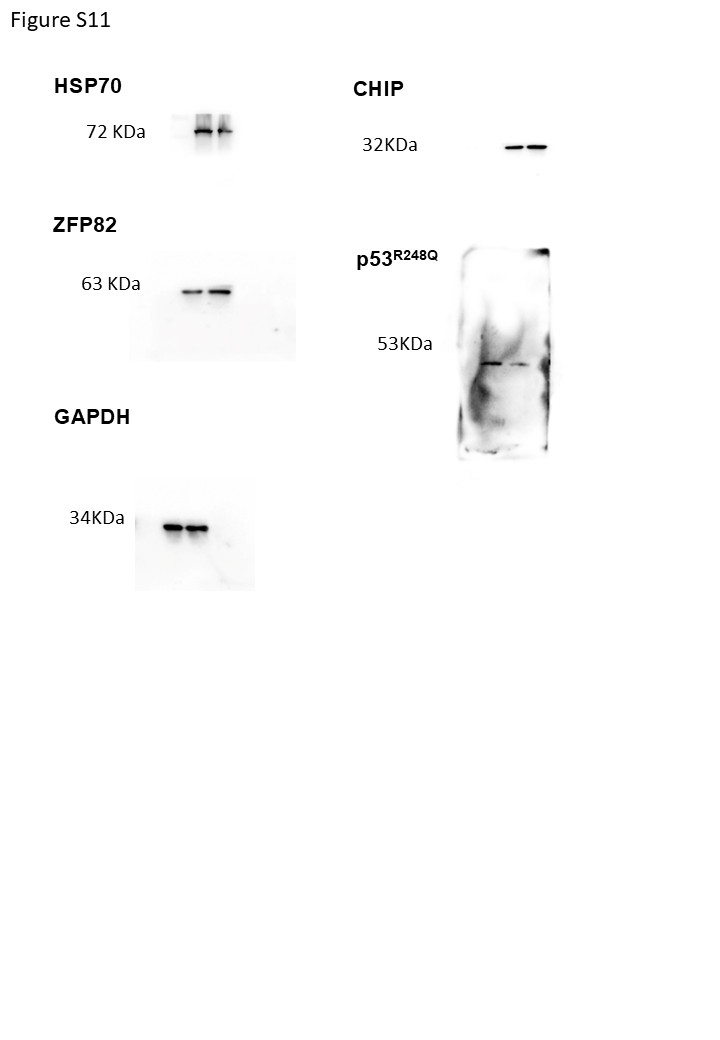

Supplement: Supplementary file 11 — Full and uncropped western blots [file 41419_2025_7979_MOESM11_ESM.jpg]

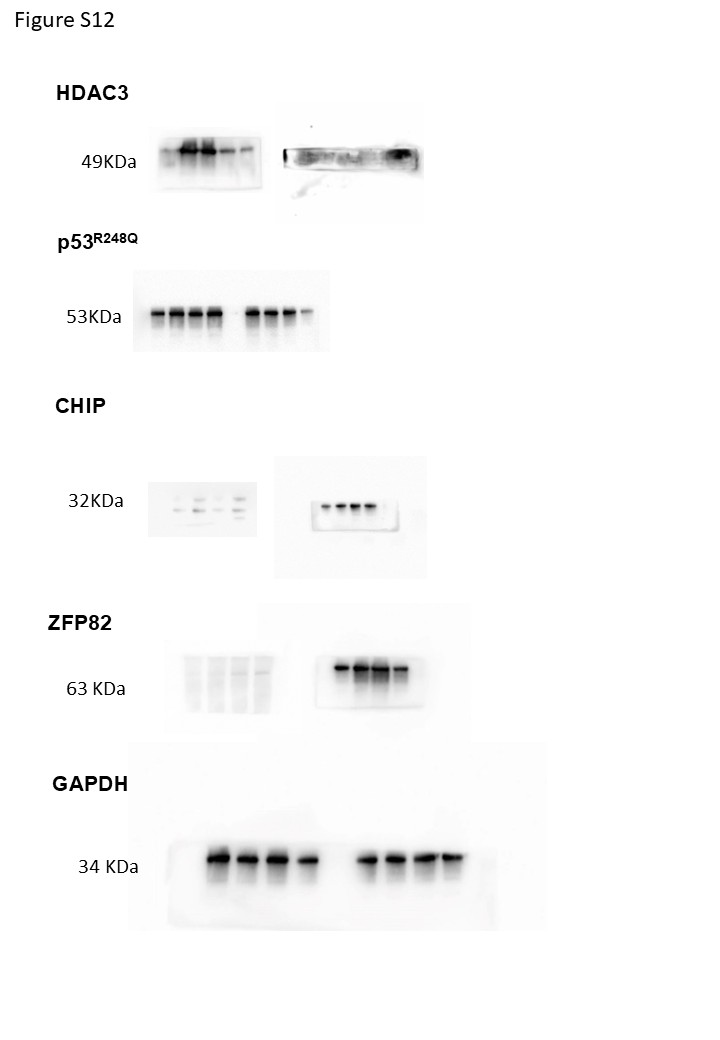

Supplement: Supplementary file 12 — Full and uncropped western blots [file 41419_2025_7979_MOESM12_ESM.jpg]
